# Human wild‐type and D76N β_2_‐microglobulin variants are significant proteotoxic and metabolic stressors for transgenic *C. elegans*

**DOI:** 10.1096/fba.2023-00073

**Published:** 2023-10-25

**Authors:** Sara Raimondi, Giulia Faravelli, Paola Nocerino, Valentina Mondani, Alma Baruffaldi, Loredana Marchese, Maria Chiara Mimmi, Diana Canetti, Guglielmo Verona, Marianna Caterino, Margherita Ruoppolo, P. Patrizia Mangione, Vittorio Bellotti, Francesca Lavatelli, Sofia Giorgetti

**Affiliations:** ^1^ Department of Molecular Medicine, Institute of Biochemistry University of Pavia Pavia Italy; ^2^ Research Department Fondazione IRCCS Policlinico San Matteo Pavia Italy; ^3^ Centre for Amyloidosis, Division of Medicine University College London London UK; ^4^ Department of Molecular Medicine and Medical Biotechnology University of Naples "Federico II" Naples Italy; ^5^ CEINGE – Biotecnologie Avanzate s.c.a.r.l. Naples Italy

**Keywords:** *C. elegans*, integrated omics, misfolding, proteotoxicity, systemic amyloidosis, β_2_‐microglobulin

## Abstract

β_2_‐microglobulin (β_2_‐m) is a plasma protein derived from physiological shedding of the class I major histocompatibility complex (MHCI), causing human systemic amyloidosis either due to persistently high concentrations of the wild‐type (WT) protein in hemodialyzed patients, or in presence of mutations, such as D76N β_2_‐m, which favor protein deposition in the adulthood, despite normal plasma levels. Here we describe a new transgenic Caenorhabditis elegans (*C. elegans*) strain expressing human WT β_2_‐m at high concentrations, mimicking the condition that underlies dialysis‐related amyloidosis (DRA) and we compare it to a previously established strain expressing the highly amyloidogenic D76N β_2_‐m at lower concentrations. Both strains exhibit behavioral defects, the severity of which correlates with β_2_‐m levels rather than with the presence of mutations, being more pronounced in WT β_2_‐m worms. β_2_‐m expression also has a deep impact on the nematodes' proteomic and metabolic profiles. Most significantly affected processes include protein degradation and stress response, amino acids metabolism, and bioenergetics. Molecular alterations are more pronounced in worms expressing WT β_2_‐m at high concentration compared to D76N β_2_‐m worms. Altogether, these data show that β_2_‐m is a proteotoxic protein in vivo also in its wild‐type form, and that concentration plays a key role in modulating pathogenicity. Our transgenic nematodes recapitulate the distinctive features subtending DRA compared to hereditary β_2_‐m amyloidosis (high levels of non‐mutated β_2_‐m vs. normal levels of variant β_2_‐m) and provide important clues on the molecular bases of these human diseases.

## Graphical abstract

The nematode Caenorhabditis elegans has been used to scrutinize the molecular complexity of protein amyloidogenesis. The combined analysis of phenotypic defects, cell stress and proteome/metabolome changes induced by the expression of human β_2_‐microblobulin provides key findings on proteotoxicity of amyloidogenic proteins. Created with BioRender.com.

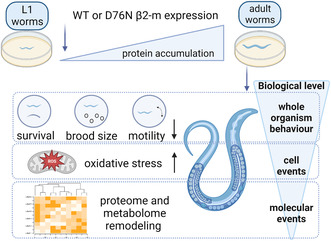

## 1 INTRODUCTION

β_2_‐microglobulin (β_2_‐m) is a paradigmatic model system useful to study the general rules dictating the amyloid conversion of globular proteins as it happens in systemic amyloidosis.^1^ β_2_‐m is the light chain of Class I major histocompatibility complex (MHCI), a membrane‐bound protein‐assembly involved in immune recognition by T cells.^2^ Unbound β_2_‐m is a well‐known amyloidogenic protein in humans, being the causative agent of β_2_‐m‐related amyloidosis (Aβ_2_‐m) in two distinct settings. The first one is dialysis‐related amyloidosis (DRA), in which the wild type (WT) form of β_2_‐m generates amyloid deposits in patients that undergo long‐term dialysis, when this protein is not efficiently cleared from serum and its concentration increases from an average of 0.16–3.2 μM.^3^ Over the years, this leads to the deposition of insoluble protein aggregates typically localized in the osteoarticular structures causing pathological bone and joint destruction. It is worth noting that concentrations of circulating WT β_2_‐m higher than normal are measured in humans not only in patients affected by chronic renal failures, but also in some pathological conditions including solid organ malignancies, lymphoproliferative disorders, and many autoimmune diseases, or in elderly people.^4^, ^5^

The second setting is represented by amyloid deposition from β_2_‐m genetic variants. The first variant of β_2_‐m, D76N β_2_‐m, discovered in a French family in 2012, is responsible for a completely different type of amyloidosis characterized by deposits in internal organs and by a late onset of clinical manifestations that occur in the fifth decade of patient life.^6^ D76N β_2_‐m aggregates into amyloid at normal serum concentrations, without involving the WT protein circulating in plasma of heterozygous individuals. In vitro, the D76N variant displays a lower thermodynamic stability and a strongly enhanced amyloidogenic tendency to aggregate compared to the WT protein.^7^ More recently, another amyloidogenic variant of β_2_‐m, V27M β_2_‐m, was identified in a Japanese hemodialyzed patient suffering from painful polyarthropathy and macroglossia, thus showing different clinical manifestations compared to D76N β_2_‐m patient.^8^

Despite the extraordinary progress achieved in the last decade in elucidating the general mechanism of β_2_‐m amyloidogenesis and the cytotoxicity mechanisms of its different aggregation states,^9^, ^10^, ^11^ a detailed understanding of pathways by which the aggregation process results in cell and tissue damage remains a challenging unsolved issue. Animal models are crucial for providing clues on proteotoxicity mechanisms and furthermore for the identification of the specific targets on which the damage occurs. So far, attempts to reproduce Aβ_2_‐m in mice have failed.^12^ In the lack of a mammalian model, however, we and others have exploited the invertebrate Caenorhabditis elegans (*C. elegans*) as a model system for recapitulating β_2_‐m toxicity in vivo.^13^, ^14^, ^15^ Although worm models cannot reproduce the clinical and pathologic complexity of the human disease, they proved to be able to highlight and single out specific steps within the process. All the *C. elegans* β_2_‐m expressing strains established so far display a pathological phenotype with reduced lifespan, impaired motility, and developmental delays.^13^, ^14^, ^15^ In particular, the production of the highly amyloidogenic D76N variant β_2_‐m both in our *C. elegans* strain,^14^ as well as in the nematode model recently described by Good et al. was shown to cause enhanced proteotoxicity.^15^ In these models WT β_2_‐m levels were comparable to those of the mutated isoforms and the expression of WT protein was linked to a mild pathological phenotype, suggesting that it is less toxic than the variants. However, the mechanism underlying the proteotoxicity is still unclear and it is not understood which are the effect on the proteome and metabolome. Therefore, first of all, we have generated stable *C. elegans* strain with the transgene for WT β_2_‐m integrated in the worm genome and a temperature‐dependent induction of protein expression as previously done for the D76N variant.^14^ Interestingly the levels of WT protein expressed in all the newly established strains were higher than those secreted in the mutant line.

The availability of two strains, one producing a highly amyloidogenic variant at lower concentrations, as occurs in hereditary Aβ_2_‐m patients, and one producing an intrinsically more stable isoform at higher concentrations, as occurs for example in hemodialysis patients, allowed us to compare the effect of WT and D76N β_2_‐m variant on the phenotype, proteome and metabolome of the corresponding *C. elegans* strains.

## 2 METHODS

### 2.1 Construction of *C. elegans* strain and maintenance

PD8120 smg‐1(cc546) *C. elegans* strain was provided by the Caenorhabditis Genetics Center (CGC, University of Minnesota, USA). The generation of the *C. elegans* transgenic strain expressing D76N β_2_‐m (named CPV27) was already described previously.^14^ In a similar manner, completely stable chromosomally integrated line expressing WT β_2_‐m was obtained after UV irradiation by SunyBiotech company (SunyBiotech Co., Ltd, Tai jiang District of Funzhou City, Fu Jian Province, China), and one clone named PHX146, was chosen for subsequent analysis. After irradiation, PHX146 strain was back‐crossed with PD8120 ancestral worms in order to remove background mutations arising from the irradiation process. The genotype of WT β_2_‐m‐expressing nematodes was confirmed by single‐worm PCR and DNA sequence analysis. The PD8120 control strain and β_2_‐m strains were grown in Petri dishes on nematode growth medium (NGM) and fed with the OP50 strain of Escherichia coli (*E. Coli*). Age synchronized worms were obtained by bleaching adult nematodes with alkaline solution (500 mM NaOH, 1.5% NaClO) and eggs were isolated and maintained at 20°C. When they reached the L1 larval stage, the expression of D76N/ WT β_2_‐m was induced by increasing temperature to 25°C. For proteomic and metabolomic analysis, nematodes were grown in liquid culture in S‐basal buffer with addition of S‐Complete components and of *E. Coli* HB101 bacteria. The flasks were maintained at 25°C under stirring at 150 rpm and bacteria were added when necessary. Once they reached the second day of adulthood, larvae were removed by filtration daily in order to obtain a homogeneous population of adult worms.

### 2.2 Gene expression analysis

RNA from adult transgenic worms was prepared using the miRNeasy Mini Kit (QIAGEN, 217004) and quantified using the NanoDrop apparatus (ThermoScientific). Total RNA was reverse transcribed into cDNA with SuperScript IV First‐Strand Synthesis System (Invitrogen, 18091050). A quantitative real‐time PCR (qRT PCR) was performed as previously described in Diomede et al.,^11^ with QuantStudio 3 PCR cycler (Applied Biosystems) using the QUANTIFAST SYBR GREEN PCR kit (QIAGEN, 204054). Relative quantification of β_2_‐m mRNA level was determined using endogenous standard gene control cell division cycle 42 (cdc‐42). All measurements were determined in triplicate. Data points collected correspond to the number of PCR cycles (Ct value) required for the fluorescent signal to cross the detection threshold of the thermal cycler. Ct values were normalized to correct for minor differences in cDNA concentrations by subtracting the average of the Ct values of the reactions in triplicate of each transgenic strain from the geometric mean of Ct values of cdc‐42 reactions and analyzed using the comparative 2^−ΔΔCt^ method.

### 2.3 Analysis of β_2_‐m expression by western blotting

Worms were collected at the first or fifth day of adulthood, in M9 buffer (45 mM KH_2_PO_4_, 42 mM Na_2_HPO_4_, 85 mM NaCl, 1 mM MgSO_4_ in water) and lysed by sonication in lysis buffer (25 mM Tris–HCl pH 7.5, 5 mM NaCl, 5 mM EDTA, 1 mM DTT, protease inhibitor cocktail Roche Applied Science). For each lysate, equal amounts of total proteins, quantified with the Pierce BCA Protein Assay Kit (ThermoScientific), were loaded onto a 4%–20% Mini‐PROTEAN TGX (Biorad) for electrophoresis performed under reducing conditions. Proteins were transferred to Immobilon P membranes (Millipore) and blocked with 5% nonfat milk, in tris‐buffered saline and Tween 20 (TBS‐T), for 1 h. Western blots were developed with 4.6 μg/mL rabbit polyclonal anti‐human β_2_‐m antibody (Agilent DAKO Cat#A0072) overnight at 4°C and 1.3 ng/mL anti‐rabbit IgG peroxidase conjugate (Sigma‐Aldrich Cat#A0545, RRID:AB_257896) for 1 h RT, as primary and secondary antibody, respectively. To normalize the content of total protein, western blot was developed with 0.185 μg/mL anti‐glyceraldehyde−3‐phosphate dehydrogenase antibody (anti‐GAPDH selected as loading control, (Abcam Cat# ab181602, RRID:AB_2630358) overnight at 4°C, and 1.3 ng/mL secondary anti‐rabbit IgG peroxidase conjugate (Sigma‐Aldrich Cat#A0545, RRID:AB_257896) antibody for 1 h RT. Immunoreactive bands were detected by ECL chemiluminescence (Millipore), and quantified with Image Studio Lite (LI‐COR Biosciences).

### 2.4 Self‐assembly of WT β_2_‐m in transgenic *C. elegans* strain

A pellet containing transgenic *C. elegans* was resuspended in M9 and lysis buffer for subsequent sonication. After centrifugation at 21,000 *g* for 10 min at 4°C, the supernatant was collected as the soluble fraction and the pellet was collected as the insoluble fraction. The insoluble pellet was washed twice in PBS buffer, resuspended in a 10% SDS solution and boiled for 10 min at 95°C. Soluble fraction and resuspended insoluble fraction were analyzed by 4%–20% SDS‐PAGE and immunoblotted as described above.

The soluble fraction was then diluted to 1 mg/mL with water and a single 500 μL sample containing 0.5 mg total protein was loaded into a Superdex 75 10/300 GL gel filtration column equilibrated and eluted with PBS pH 7.5 at a flow rate of 0.5 mL/min using an Akta Pure FPLC. Fractions of 1 mL were collected and analyzed by 4%–20% SDS‐PAGE and immunoblotted as above.

### 2.5 Characterization of *C. elegans* phenotype

#### 2.5.1 Larval growth and motility automated assay (INVAPP/Paragon system)

The investigation of the effect of expressing β2‐m on nematode growth and motility was performed by using INVAPP/Paragon system as reported in Faravelli et al.^14^ Three synchronized nematodes, at their L4 larval stage, were picked onto NGM plates and incubated at 25°C for 4 days after reaching adulthood. Plates were imaged using a stereo‐microscope (M165FC Leica) coupled to a digital microscope camera (Leica DFC425C) at a magnification 0,5X in order to image total area of each plate. Movies were captured using μManager and analyzed with a set of MATLAB (MATLAB, RRID:SCR_001622) scripts (https://github.com/fpartridge/invapp‐paragon). Briefly, this involved calculating the variance through time for each pixel. Pixels whose variance was above the threshold (typically those greater than one standard deviation away from the mean variance) were considered “motile.” The “motile” pixels were counted, and a movement score was generated for each plate. The data presented as mean ± SEM were tested for significance by the nonparametric Mann–Whitney test, using GraphPad Prism (RRID:SCR_002798). Significant results were marked according to critical *p*‐values: ***p < 0.001; **p < 0.01; *p < 0.05.

#### 2.5.2 Motility automated assay (INVAPP/Paragon system)

Worms were synchronized and grown at 25°C on NGM plates until they reached the Day 5 of adulthood. A total number of 50 worms per each strain were randomly picked and put into drops of M9 containing 10 worms per each drop for the motility evaluation. Through the INVAPP‐Paragon automated system, 200 frames were captured using μManager and analyzed with the MATLAB scripts as above. The data presented as mean ± SEM were tested for significance by the nonparametric Mann–Whitney test, using GraphPad Prism. Significant results were marked according to critical *p*‐values: ***p < 0.001; **p < 0.01; *p < 0.05.

#### 2.5.3 Life‐span assay

Forty synchronized adult worms maintained at 16°C were upshifted to 25°C at the larval stage L1. Every day, they were transferred onto a freshly prepared NGM plate until the cessation of egg‐laying to avoid the overlapping of generations. Viability was monitored until all worms were reported dead when they failed to display touch‐evoked movement. The data were tested for significance in the log‐rank Mantel–Cox test, using GraphPad Prism. Significant results were marked according to critical *p*‐values: ***p < 0.001; **p < 0.01; *p < 0.05.

### 2.6 Superoxide production

Live nematodes were dispensed into wells of a 96‐well plate containing Amplex UltraRed reagent (Life Technologies, final concentration 0.1 μM in M9 buffer). Samples were incubated at 25°C for 3 h, thus fluorescence was read in a BMG LABTECH FLUOstar Omega plate reader at excitation 544 nm and emission 590 nm. Data were normalized based on the total proteins content of each sample. The data presented as mean ± SEM were tested for significance by the nonparametric Mann–Whitney test, using GraphPad Prism. Significant results were marked according to critical *p*‐values: ***p < 0.001; **p < 0.01; *p < 0.05.

### 2.7 Proteomic analysis

#### 2.7.1 Sample preparation for proteomic analysis

Synchronized worms recovered at day 5 of adulthood were lysed by sonication in lysis buffer (25 mM Tris–HCl pH 7.5, 5 mM NaCl, 5 mM EDTA, 1 mM DTT, protease inhibitor cocktail Roche Applied Science). Following proteins extraction by chloroform/methanol/water precipitation, three dry extracts of about 3000 worms for each strain were digested in TEZ buffer (10 mM Tris–HCl pH 8, 1 mM EDTA, 0.2% Rapigest) by heating at 99° for 20 min. After centrifugation at 20817 *g* for 5 min and sonication for 15 min at room temperature the samples were treated with trypsin (Trypsin Gold Mass Spectrometry Grade, Promega) and incubated overnight at 37°C. Peptide mixtures were then reduced with dithiothreitol at 99°C for 5 min.^16^

#### 2.7.2 Data acquisition and processing

Proteomics data acquisition was performed on a Thermo ScientificTM Q‐Exactive Plus Orbitrap mass spectrometer connected to an Ultimate 3000 nanoLC system. Samples were trapped on a Thermo Scientific Acclaim PepMap C18 cartridge (0.3 mm × 5 mm, 5 μm/100 Å) and then chromatographed on a Thermo Scientific Easy‐Spray Acclaim PepMap C18 column (75 μm × 15 cm, 3 μm/ 100 Å packing) with a 41‐min linear gradient of acetonitrile:water:formic acid (3:97:0.1–44:56:0.1 v/v/v) at 300 mL/ min.

A full MS scan (m/z 350–1400) was acquired with a maximum injection time of 100 ms, and the 10 most intense ions were selected for higher energy C‐trap dissociation (HCD). The normalized collision energy was set to 28, with an isolation width of 2 Da and dynamic exclusion of 20 s; singly charged ions were excluded.

A total number of 27 liquid chromatography–tandem mass spectrometry (LC–MS/MS) raw files, three technical replicates for each biological sample, were processed using MaxQuant (version 2.0.1.0) for protein identification and quantification according to the MaxLFQ algorithm.^17^ Data were analyzed with the Andromeda search engine against the freely available reference proteome of *C. Elegans* (Organism ID:6239; Proteome ID: UP000001940; total proteins: 26,584) downloaded from the UniProtKB database (October 2021) including a list of common contaminants.^18^ The precursor and the fragment mass tolerance were set to 4.5 and 20 ppm, respectively. The minimum peptide length was set to eight amino acids and trypsin was selected as proteolytic enzyme, allowing up to two missed cleavage sites. Oxidation (Met), N‐term acetylation and N‐terminal glutamate to pyroglutamate conversion were set as variable modifications. The false discovery rate (FDR) at both the protein and peptide level was set to 1%. The mass spectrometry proteomics data have been deposited to the ProteomeXchange Consortium that is available at http://www.proteomexchange.org (RRID:SCR_004055), via the PRIDE partner repository with the dataset identifier PXD040230. *Reviewer account details*: *username*: reviewer_pxd040230@ebi.ac.uk *Password*: *YMl10EDj*.

#### 2.7.3 Statistical analysis

A statistical analysis of the MaxQuant results was performed using the Perseus software platform (version 1.6.15.0). The dataset was filtered removing proteins only identified by site, potential contaminants and reverse hits. Label‐free quantification (LFQ) intensities were log2 transformed and further filtering was carried out considering for each protein valid LFQ values for two out of three replicates in at least one of the three groups. Missing values were imputed as simulated values forming an ideal Gaussian distribution (width = 0.5; down shift = 1.8).

An ANOVA statistical test was first conducted on filtered and log2 transformed LFQ intensities after Z score normalization. Hierarchical clustering was then performed using the Euclidian distance and the average linkage and results were displayed in a visual heat map.

The binary comparison between proteomic profiles of transgenic *C. elegans* strains and the control was carried out on significant proteins according to a Student’ s *t*‐test (S0 = 0.1 and FDR = 0.05) and 1.3 was set as minimum fold change. The difference between the average log2 LFQ intensities for each strain were used to calculate the differential abundance.

#### 2.7.4 Pathway analysis

STRING (v. 11.5) bioinformatic tool was used to perform pathway enrichment analysis on proteins with differential abundance in the two transgenic nematodes strains in comparison with the control strain. The full STRING network, based on both functional and physical protein association, was taken into account for the analysis and a minimum confidence score of 0.9 was required for protein–protein interaction identification.

### 2.8 Metabolomic analysis

#### 2.8.1 Sample preparation for metabolomic analysis

Three samples of approximately 12,000 synchronized adult worms at day 5 of adulthood for each strain were resuspended in a MeOH/H_2_O extraction solvent (50/50% v/v) and homogenized in a tissue‐lyser (Minilys, Bertin Instruments) with 4 cycles of 10 s interleaved with 2 min pauses for the chilling of vials in dry ice. After centrifugation at 21,000 *g* for 20 min at 4°C, the supernatant containing hydrophilic metabolites was divided into six aliquots which were then dried with a speed‐vac (2 h at RT) and stored at −20°C.

Four aliquots of each sample were directly used for NMR analysis after redissolving and one aliquot was instead further processed prior to the mass spectrometry analysis following protocols of MxP® Quant 500 kit (Biocrates Life Sciences AG, Innsbruck, Austria). Specifically, dry supernatants were first solubilized in 12 μL MeOH and 10 μL loaded onto the MxPQuant 500kit plate. After drying under nitrogen stream, they underwent a derivatization reaction consisting in 1 h incubation with 5% PITC (phenylisotiocyanate) in 50 μL of EtOH/H_2_O/Pyridyne 1:1:1 v/v/v. Samples were nitrogen dried and then subjected to an additional extraction step of 30 min shaking after addition of 300 μL of 5 mM ammonium acetate in MeOH. The extracts were eluted by centrifugation for 2 min at 500 *g* and the extracted material (150 μL) was diluted with 100 μL of HPLC‐grade water.

#### 2.8.2 NMR Spectra acquisition and processing

Aliquots of dried extract were redissolved in 0.58 mL of deuterated phosphate buffer at pH 7.4, containing: Na_2_HPO_4_/NaH_2_PO_4_ 50 mM and TSP‐Na^+^‐d4 (sodium 3‐trimethyl‐silyl[2,2 3,3‐d4] propionate) 0.2 mM as a frequency reference.

The NMR acquisition started no more than 30 min after sample reconstitution; meanwhile the samples were kept on ice. The NMR working temperature was 298 K. NMR spectra were acquired on a Bruker Avance NEO 700 MHz spectrometer equipped with a TCI CryoProbe Triple resonance and with single axis Z‐gradient. For each sample a 1D‐1H NMR spectrum was acquired for quantitative estimation of metabolites. A set of two‐dimensional NMR spectra was also acquired to enable the molecular identification and validation of 1D NMR peaks assignment. Experimental details are reported in Supporting Information section.

The 1D NMR spectra were integrated by spectral bucketing, that is, the processed 1D NMR spectra were divided into stripes of 0.01 ppm width and each stripe's area was integrated, after exclusion of empty regions containing impurities (1.16–1.21 ppm; 3.63–3.68 ppm) and H_2_O residual signal (4.66–4.95 ppm). The result was a data matrix of bucket area of nine samples (rows) x 956 chemical shifts (columns). Each area was normalized against the total spectral area, in order to remove the samples concentration inhomogeneity, and was used to evaluate the relative level of each metabolite among the three worm lines.

#### 2.8.3 NMR peaks assignment

The assignment of peaks of 1D NMR spectra to metabolites relied, substantially, on database query such as BMRB (Biological Magnetic Resonance Bank) (https://bmrb.io/metabolomics/) and The Human Metabolome Database (HMDB) (https://hmdb.ca/), or on literature^42^, ^43^ and was confirmed by two‐dimensional NMR (2D NMR) spectra.

#### 2.8.4 Multivariate statistical analysis of NMR output dataset

Unsupervised multivariate statistical approach, as principal components analysis (PCA) and hierarchical cluster analysis, were applied prior to assignment on the matrix of 1D NMR data to explore clustering patterns of samples, trends in the data and potential outliers. The web tool MetaboAnalyst 5.0 (https://www.metaboanalyst.ca/, RRID:SCR_015539) was used.

PCA was performed after row‐wise normalization of bucket area against the total spectral area and column‐wise scaling by Pareto algorithm.

Hierarchical cluster analysis was performed selecting Euclidean distance as similarity measure parameter and Ward's linkage as clustering method (to minimize the sum of squares of any two clusters) as clustering algorithm.

#### 2.8.5 Univariate statistical analysis of NMR output dataset

A total of 36 metabolites was unambiguously identified and quantified. For each metabolite we selected the peak with the best signal/noise ratio and with no overlapped signals.

With the resulting selected dataset of normalized bucket area, we performed an unpaired *t* test, correcting for multiple comparison by FDR with two‐stage step‐up method,^44^ and fixing the desired FDR (Q) value at 1%. The test was performed using GraphPad Prism Version 9.2.0.

This test, an analogous of t test for samples with three or more groups, allowed us to detect the metabolites which were significantly different among the three *C. elegans* strains.

#### 2.8.6 MS data acquisition and processing

LC‐MS/MS in multiple‐reaction monitoring (MRM) mode was used to target and quantitate metabolites. For each sample three technical replicates were carried out on a Triple Quad™ 5500+ System – QTRAP® Ready (AB Sciex) coupled to a 1260 Infinity II HPLC (Agilent) for the liquid chromatography. Data were generated by the Analyst software v.1.7.1 (AB Sciex) and metabolites concentrations were calculated using the MetIDQ™ Oxygen software (Biocrates Life Sciences AG).^19^

#### 2.8.7 Statistical analysis of MS output dataset

The concentration values of the identified metabolites were imputed to remove missing values and normalized by sum using MetaboAnalyst 5.0 software (each metabolite concentration was divided to the sum of concentrations of all metabolites in each sample) in order to overcome samples' inhomogeneity.

The nonparametric Mann–Whitney test was applied, correcting for multiple comparison, to simultaneously compare the two β_2_‐m expressing strains to the ctrl one. The test was performed for each class of quantified metabolites and differences were considered significant when q values were below 0.01. GraphPad Prism version 9.0 software was employed.

### 2.9 Integrated pathway analysis of proteomics and metabolomics data

All significantly altered proteins and metabolites, resulting from both NMR and MS analysis, were pooled into a single query and subjected to a joint pathway analysis through Metaboanalyst 5.0 software. *C. elegans* Kyoto Encyclopedia of Genes and Genomes (KEGG) database was interrogated to find enriched pathways. The pathway impact was evaluated according to degree centrality, which measures the number of links that connect each node (metabolites or protein) to the other nodes within the pathway network.

## 3 RESULTS

### 3.1 Novel WT β_2_‐m thermoinducible transgenic *C. elegans* strain

**FIGURE 1:**
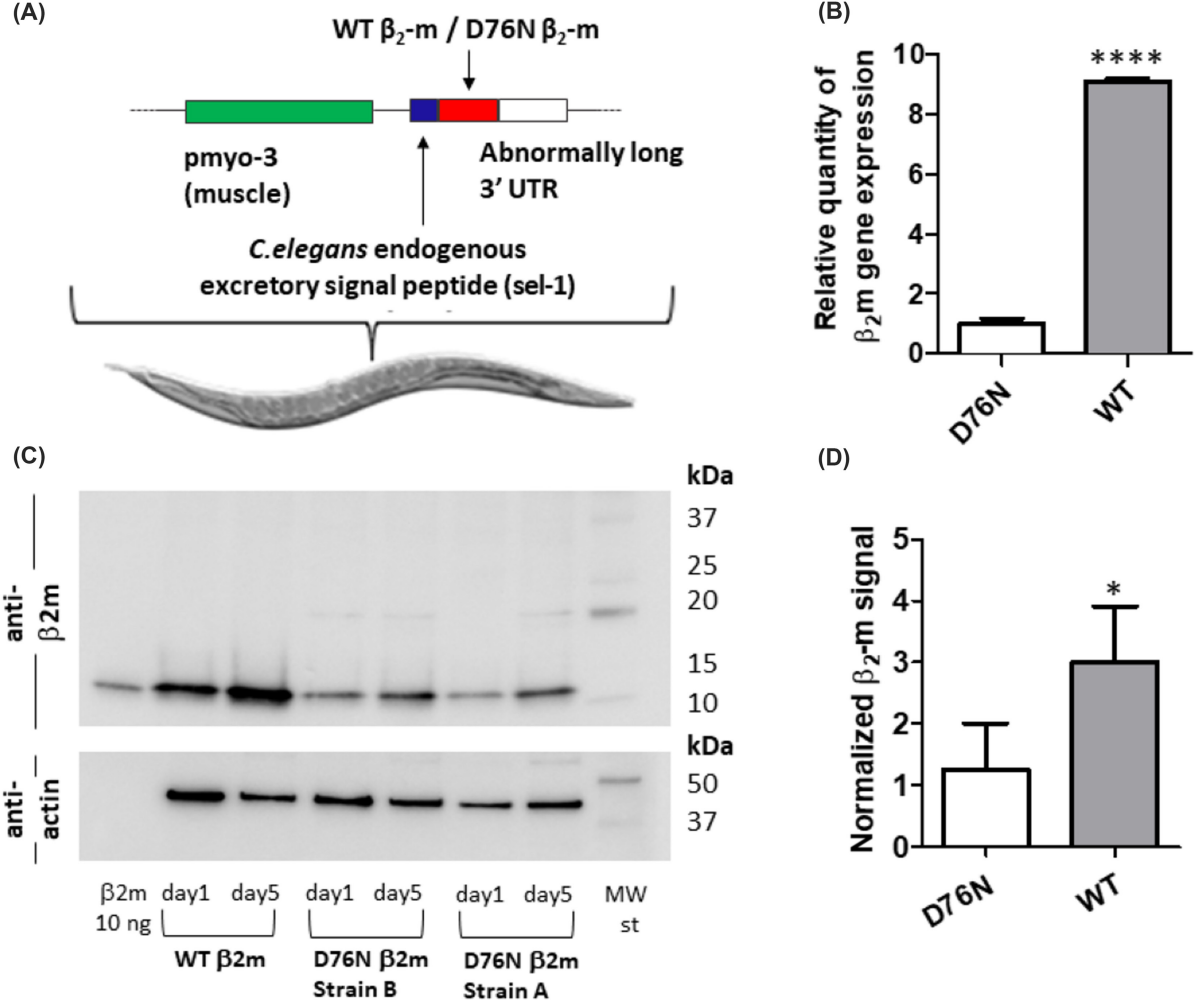
(A) Scheme of chromosomally stable integrated *C. elegans* strains expressing WT β_2_‐m and the highly amyloidogenic variant D76N β_2_‐m.^14^ Both the isoforms were expressed under the temperature inducible control of the body‐wall muscle–specific promoter myo‐3 with the signal sequence of the endogenous excretory signal peptide sel‐1. (B) Strain genotyping characterization. Human β_2_‐m mRNA expression in the two transgenic strains was normalized relative to cdc 42 (cell division cycle 42, GTP‐binding protein) mRNA, as endogenous reference. Data are expressed as mean and SEM (bars) of two independent experiments, ****p  < 0.0001 for WT versus D76N according to Mann–Whitney test. (C) Representative western blots showing β_2_‐m protein levels. 10 μg of total proteins extracted from D76N (Strain A and Strain B) or WT β_2_‐m nematodes recovered at day 1 and Day 5 of adulthood were loaded on gel and immunoblotted using polyclonal anti‐human β_2_‐m antibody (M = molecular weight standard: Precision Plus Western C, BioRad). Uncropped scans of immunoblot are shown in Supplementary material (Figure S2A). Strain A of D76N β_2_‐m nematodes was used for all the other analysis reported in the paper. (D) Normalized β_2_‐m protein levels, indicated as β_2_‐m/actin ratio of the WB band density in nematodes at 5 days of adulthood. Four independent WB experiments were carried out and the results were plotted as mean and SEM (bars) using GraphPad Prism v5, *p < 0.05 for WT versus D76N according to Mann–Whitney test.

Transgenic *C. elegans* were engineered to express WT β_2_‐m in the body‐wall muscles under the control of the myosin promoter (myo‐3), by using the smg temperature‐dependent expression system.^14^ The endogenous sel‐1 protein signal sequence was included (Figure 1A) to enable β_2_‐m secretion from muscles and mimic the natural extracellular location of the protein.

The WT β_2_‐m‐expressing strain was selected among six WT β_2_‐m strains originally generated in which the levels of β_2_‐m expression were higher than those observed in the previously established D76N β_2_‐m worms (data not shown).

Although the transgenes copy number was the same in the two strains, as shown by quantitative PCR (qPCR) on genomic β_2_‐m coding sequence (Figure S1), the transcriptional expression levels of the two β_2_‐m isoforms were different. The β_2_‐m mRNA levels were measured on lysates of worms at the second day of adulthood grown at 25°C from L1 larval stage by qRT PCR (Figure 1B). mRNA levels of WT β_2_‐m in the new strain were approximately ninefold higher than that of D76N β_2_‐m strain. Currently, we have no evidence to hypothesize if this difference in transcriptional level is a serendipitous event, possibly related to the transgene insertion mode, or if it is mechanistically related to properties of the transgene or of the protein itself.

In order to verify if WT β_2_‐m protein levels were also increased, the worm lysates were analyzed by western blotting using a polyclonal anti‐human β_2_‐m antibody (Figure 1C). As shown in Figure 1D, WT β_2_‐m protein levels were on average 3.45‐fold higher than those of the D76N β_2_‐m variant at day 5 of nematode adulthood grown at 25°C. Moreover, in line with what already observed in D76N β_2_‐m nematodes,^14^ the WT β_2_‐m protein levels increased with aging from day 1 to day 5 of adulthood with temperature upshifting to 25°C from the first larval stage.

**FIGURE 2:**
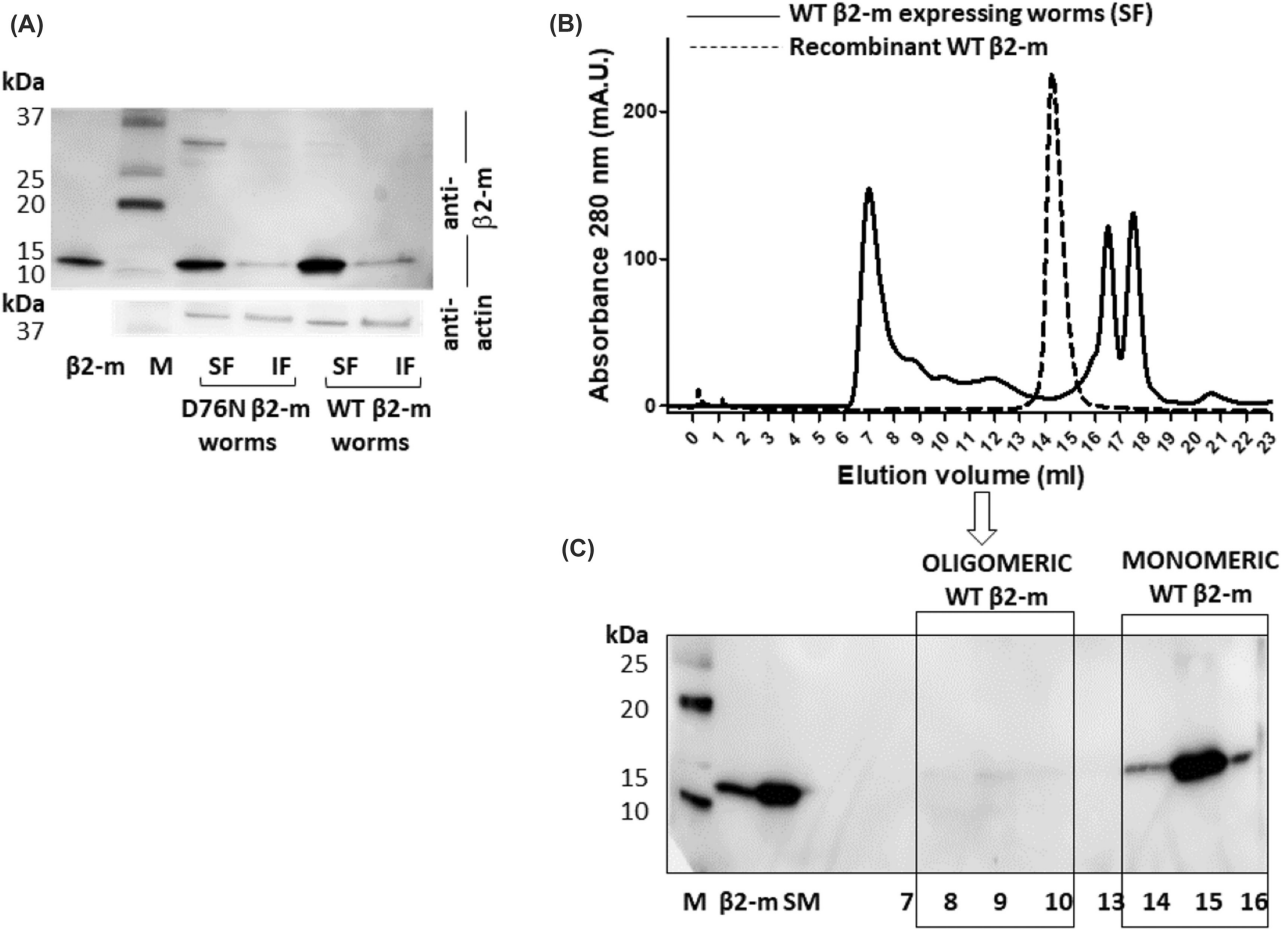
Self‐assembly of WT β_2_‐m in transgenic *C. elegans*. (A) Immunoblot analysis of soluble (SF) and insoluble fraction (IF) of worm lysates grown at 25°C, separated by 4%–20% SDS PAGE and detected with anti‐β_2_‐m antibody (DAKO). The insoluble fraction was washed twice in PBS buffer before loading onto gel. (M = molecular weight standard: Precision Plus Western C, BioRad; β_2_‐m = 20 ng β_2_‐m loaded as positive control). (B) Size‐exclusion chromatography of soluble proteins from WT β_2_‐m expressing nematodes (solid line) and recombinant WT β_2_‐m (dashed line). Fractions (1 mL) were collected. (C) Immunoblot analysis of size‐excluded fractions (7‐10, 13‐16) of β_2_‐m expressing worms' lysates resolved via 4%–20% SDS PAGE and detected with anti‐β_2_‐m antibody (DAKO). (M = molecular weight standard: Precision Plus Western C, BioRad; SM = starting material loaded into gel filtration column; β_2_‐m = 15 ng β_2_‐m loaded as positive control). Uncropped scans of immunoblots are shown in Supplementary Information (Figure S2B,C).

To investigate the aggregation state of β_2_‐m in the nematodes, soluble and insoluble fractions from the worm lysates were analyzed by western blotting (Figure 2A), showing that both WT β_2_‐m and D76N β_2_‐m were almost entirely recovered in the soluble fraction. On the contrary, the amount of β_2_‐m in the insoluble pellet was very low thus confirming our previous experimental evidence.^14^ As we had previously observed that the D76N β2‐m variant self‐aggregates in vivo generating soluble, high molecular weight, oligomeric species,^14^ we investigated whether soluble aggregates were also present in the WT β_2_‐m expressing strain. For this purpose, the soluble fraction of WT β_2_‐m nematodes was analyzed by size exclusion chromatography, followed by western blot analysis of eluted fractions (Figure 2B,C). Data showed that soluble WT β_2_‐m from the worms' lysates was eluted mainly as a monomer (fractions 14–15–16, Figure 2B,C); however, faint immunoreactive bands were also detected in higher molecular weight fractions (fractions 8–9‐10, Figure 2B,C), suggesting the presence of low amount of instable soluble oligomeric forms in accordance with the results reported for D76N β_2_‐m worms by Faravelli et al.^14^

### 3.2 Effects of β_2_‐m isoforms expression on the *C. elegans* phenotype

**FIGURE 3:**
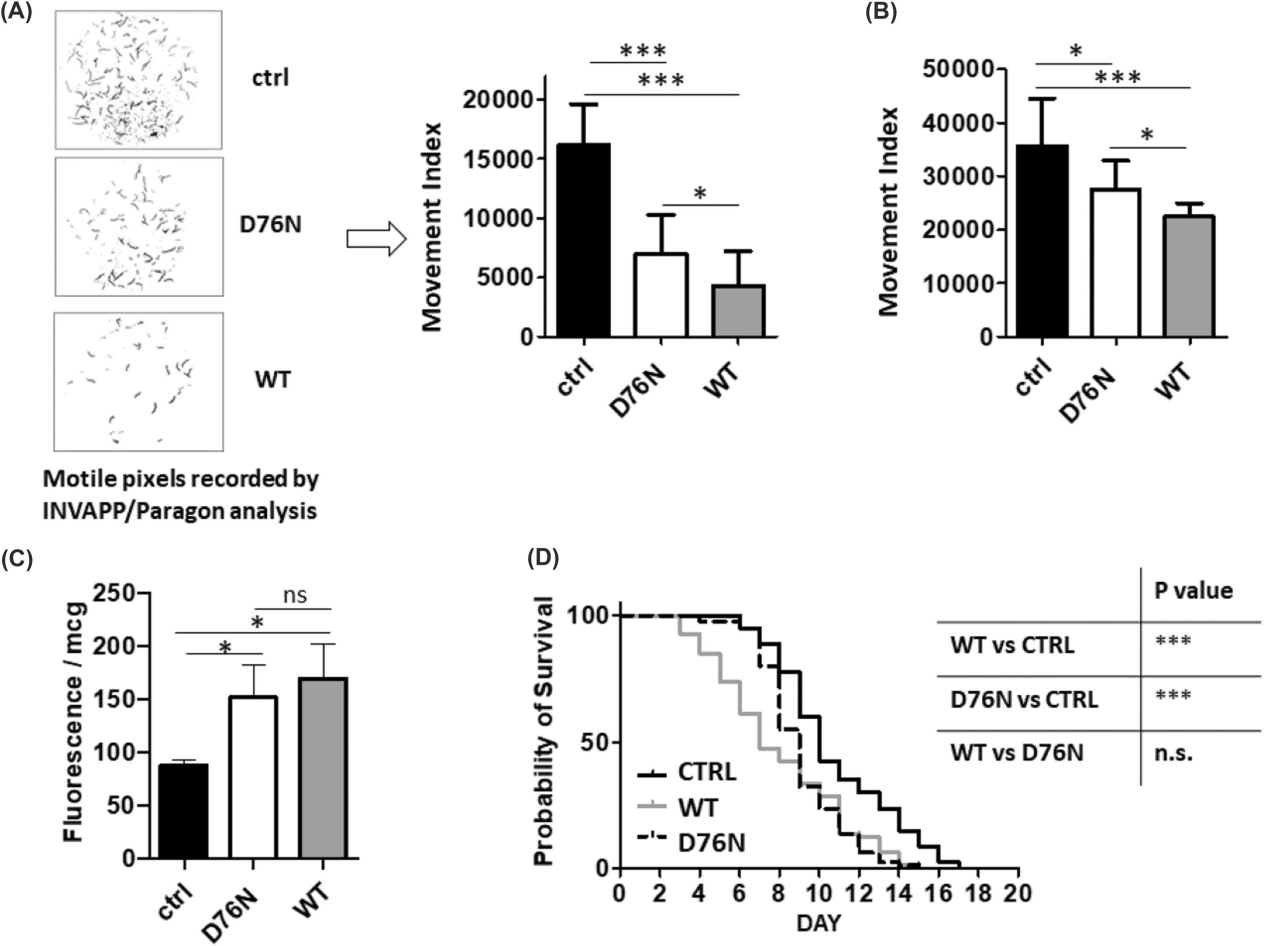
Behavioral phenotypes of transgenic *C. elegans* strains. (A) INVAPP/Paragon motility thresholding of plates containing progeny of three L4 worms grown at 25°C for 4 days after reaching adulthood. Black pixels show those scored as being “motile” by the software (left). The MI parameter, which is dependent on the number of progenies on the plate and on their movement, is then calculated from the processing of the captured movies (right) (*n* = 12 replicates per strain, each replicate was on different plates, over three experimental sessions). Data are expressed as mean and SEM (bars). The significant differences were evaluated performing nonparametric Mann–Whitney test (*p < 0.05, **p < 0.01, ***p < 0.001). (B) Motility of 10 synchronized adult nematodes at day 5 of adulthood was scored in liquid by INVAPP/Paragon system. The MI parameter, which is dependent on nematodes' movement, is calculated from the processing of the videos (*N* = 100 animals for each strain, over two experimental sessions). Data are expressed as mean and SEM (bars). The significant differences were evaluated performing non‐parametric Mann—Whitney test (*p < 0.05, **p < 0.01, ***p < 0.001). (C) The production of ROS species was measured by incubating adult worms, at day 5 of adulthood at 25°C, with Amplex UltraRed (ThermoFisher) fluorescent dye. Results show superoxide production calculated as dye fluorescence/mcg of proteins. Data are expressed as mean and SEM (bars). The significant differences were evaluated performing nonparametric Mann–Whitney test (*p < 0.05, **p < 0.01, ***p < 0.001). (D) Kaplan–Meier survival curves of adult nematodes. Animals were placed in plates seeded with OP50 starting from L4, cultured at 25°C and transferred to fresh plates for each consecutive other day. Survival rate was scored every day and expressed as percent of survival. Plots are representative of two independent experiments with *N* = 40 animals (***p < 0.0001, chi‐square 22.41 D76N versus ctrl, ***p < 0.0001, chi‐square 19.85 WT versus ctrl, ns, chi‐square 0.8671 D76N versus WT according to log‐rank Mantel–Cox test. All statistical tests performed using GraphPad Prism v5).

In order to investigate the effects of the expression of WT β_2_‐m on nematode growth and motility, and to compare it with those of D76N β_2_‐m, an automated analysis was carried out using the INVAPP/Paragon system, with calculation of the movement index (MI).^14^, ^20^ This experimental setting allows evaluating the phenotype based on movement quantification, which is dependent on the number of progenies and on their movement. Non‐transgenic nematodes of the ancestral strain, smg‐1, grown under the same conditions, were used as reference throughout the studies. In the first experiment, we followed at the same time the growth and motility of the progeny of three L4 larvae; measurements were carried out at day 5 of adulthood at 25°C (Figure 3A). Both WT β_2_‐m and D76N β_2_‐m worms strains showed lower MI than controls; MI was lower in WT β_2_‐m worms compared to D76N β_2_‐m ones (MI for WT β_2_‐m and D76N β_2_‐m worms was, respectively, 26.80% and 43.23% of the value observed for the smg‐1 control strain) (Figure 3A).

In a second type of experiment, we evaluated, using the INVAPP/Paragon system, the locomotion activity of β_2_‐m adult worms in liquid. For this purpose, 10 synchronized worms per strain at day 5 of adulthood were picked and dispensed in 90 μL of M9 buffer, followed by immediate measurement of their movement. A significant reduction of motility, compared to the ancestral control worms, was observed in both transgenic strains. In particular, we observed that the movement index for WT β_2_‐m and D76N β_2_‐m worms was, respectively, 63% and 77% of the value registered for the control strain (Figure 3B). Given the established evidence that expression of (or exposure to) several amyloidogenic proteins is associated with oxidative stress in *C. elegans* ^13^, ^21^, ^22^ we tested whether this was the case also in our transgenic nematodes, by determining superoxide production in β_2_‐m worms at the fifth day of adulthood grown at 25°C. Indeed, superoxide levels rose significantly in both transgenic strains compared to controls (Figure 3C).

Finally, in order to determine if β_2_‐m isoforms affected the nematodes' lifespan, overall survival was evaluated. Both the D76N and WT β_2_‐m expressing strains showed a significantly reduced median survival, respectively, of 9 and 7 days, compared to 10 days in controls (Figure 3D).

### 3.3 Effects of β_2−_m expression on *C. elegans* proteome

In order to explore the global effects that transgenic expression of human β_2_‐m exerts on the nematode, we compared the three strains by performing a shotgun label‐free proteomic analysis. Three replicates per strain were included in the study. Protein extracts were analyzed by LC‐MS/MS on a Q‐Exactive Plus mass spectrometer. Raw data were processed by MaxQuant according to LFQ approach and data analysis was performed using Perseus platform. A total of 1796 identified proteins were revealed and among them 1493 were correctly quantified with LFQ intensities values. To investigate the differential protein expression profiles two statistical tests (ANOVA test and Student's *t*‐test) were employed.

**FIGURE 4:**
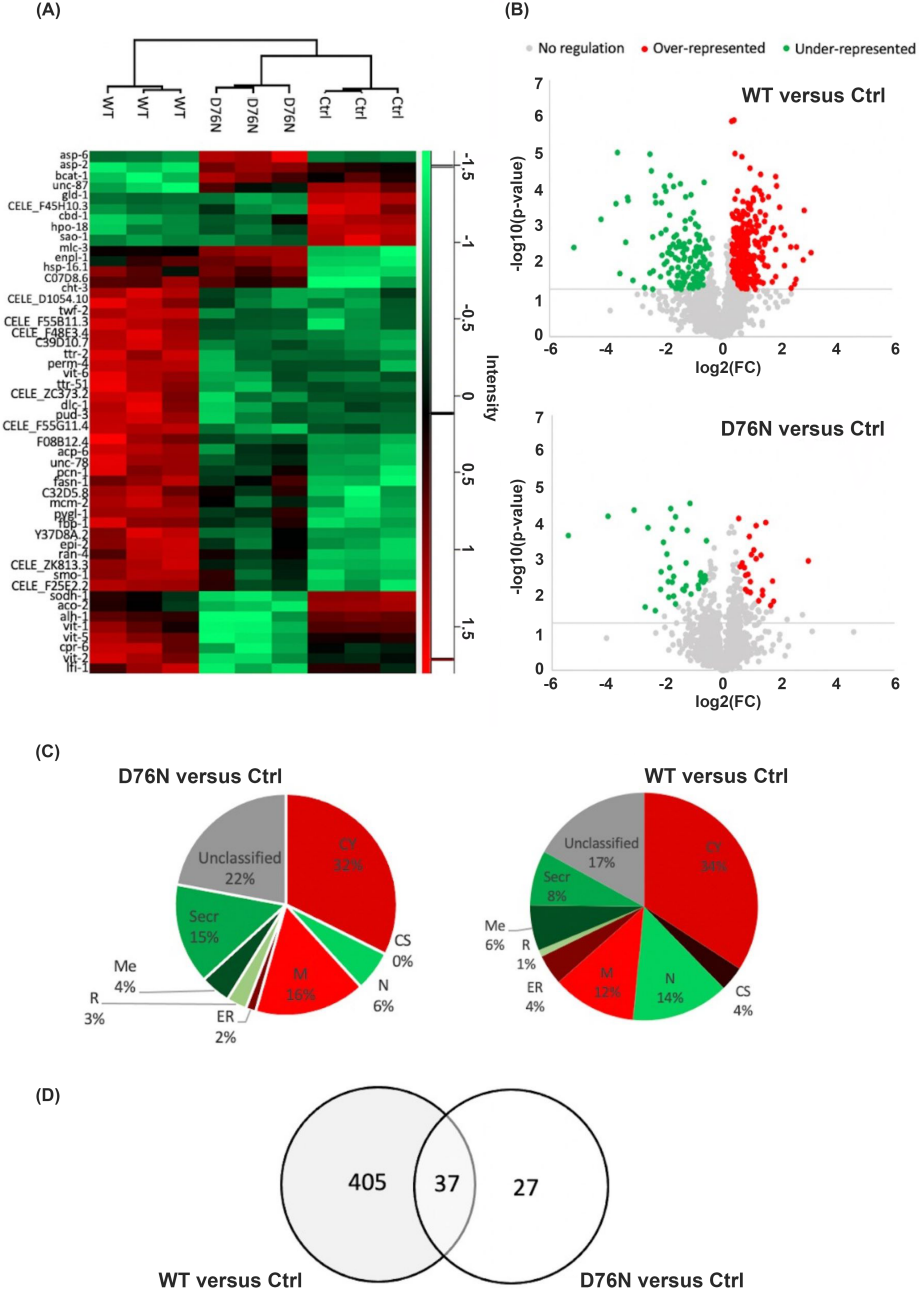
Effect of β_2_‐m isoforms expression on the nematode's proteome. (A) Results from multivariate statistical analysis, hierarchical clustering. Clustering of samples is shown with a dendrogram (top of the panel) combined with a heatmap of the top 50 ANOVA differentially abundant proteins. Heatmap colors are based on the Z‐scored (log2) LFQ values. Green shades correspond to a low relative abundance and red shades to high relative abundance. (B) Results from univariate statistical comparison of each β_2_‐m expressing strain versus control. Red and green dots represent, respectively, the proteins significantly more abundant and less abundant in the two β_2_‐m expressing strains compared to the control strain. Horizontal gray lines denote p value cutoff of 0.05. (C) *C. elegans* proteomic profile changes with β_2_‐m expression C) Subcellular location of overrepresented and underrepresented proteins in warms expressing β_2_‐m in comparison with ctrl strain. Proteins without a Gene Ontology or Uniprot annotations for cellular location are shown as ‘Unclassified’. CY, cytoplasm; N, nucleus; M, mitochondrion; CS, cytoskeleton; R, ribosome; ER, endoplasmic reticulum; Me, cell membrane; Secr., secreted. (D) Venn diagram summarizing the identified differentially represented proteins. The proteins specific for each comparison (D76N vs. ctrl, WT vs. ctrl) and those shared by the two comparisons.

The comparison across the three groups showed that 532 proteins were significantly different in abundance. The 50 most statistically relevant ones are displayed in the heatmap in Figure 4A. Clear discrimination between the three strains is visible, with greater separation of the WT β_2_‐m cluster than of the D76N β_2_‐m one from the control strain. Results of the pairwise comparison between each transgenic strain and control worms are shown in the volcano plots of Figure 4B and Table S1. The number of differential proteins is considerably higher for the comparison “WT β_2_‐m versus control nematodes” (*n* = 440 proteins, of which 300 over‐ and 140 underrepresented) than for “D76N β_2_‐m versus control nematodes” (*n* = 62 proteins, of which 24 over‐ and 38 underrepresented). According to Gene Ontology (GO) and Uniprot annotations, the differential proteins in the two transgenic strains are located in various compartments (Figure 4C). Most species are cytosolic (34% and 32% for “WT β_2_‐m vs. control” and for “D76N β_2_‐m vs. control”, respectively), however both comparisons also showed changes of proteins that are located into mitochondria, nucleus, endoplasmic reticulum, and cell membrane. In addition, several extracellular species displayed differential abundance (8% and 15% for “WT β_2_‐m vs. control” and for “D76N β_2_‐m vs. control”, respectively), including proteins belonging to extracellular matrix.

Notably, 37 proteins were differentially represented both in the “WT β_2_‐m versus ctrl” and in the “D76N β_2_‐m versus ctrl” comparisons (Table 1 and Figure 4D). Of these common proteins, all but two had the same direction of change (Table 1). Exceptions were two secreted proteins, vitellogenin−2, and vitellogenin‐5, which were downrepresented in “D76N β_2_‐m versus ctrl” and overrepresented in “WT β_2_‐m versus ctrl.”

**TABLE 1:** Differentially represented proteins shared by both D76N versus control and WT versus control comparisons.

Accesion	Protein	Gene	Localization	log2FC		*p*‐value	
				D76N versus ctrl	WT versus ctrl	D76N versus ctrl	WT versus ctrl
*Significantly over‐represented proteins shared by both D76N versus ctrl and WT versus ctrl comparisons*							
P02513	Heat shock protein Hsp‐16.48/Hsp‐16.49	hsp‐16.48	Unclassified	1.73	1.553	3.52E‐03	2.28E‐03
Q20311	ML domain‐containing protein ATP synthase subunit epsilon, mitochondrial	CELE_F42A10.6	Unclassified	1.665	1.268	1.64E‐02	3.21E‐02
P34696	Heat shock protein Hsp‐16.1/Hsp‐16.11	hsp‐16.1	Unclassified	1.496	1.358	8.78E‐05	3.54E‐03
O44952	Lon protease homolog, mitochondrial	C34B2.6	M	1.379	1.049	8.52E‐03	2.83E‐02
P55216	Putative cystathionine gamma‐lyase 2	cth‐2	CY	1.376	0.707	6.34E‐03	4.88E‐03
Q20660	SHSP domain‐containing protein	hsp‐17	Unclassified	1.327	0.695	6.82E‐04	9.31E‐03
Q20062	Isochorismatase domain‐containing protein	marb‐1	CY	1.243	1.246	1.23E‐02	6.39E‐04
P53014	Myosin, essential light chain	mlc‐3	CY	1.151	0.696	1.05E‐04	6.20E‐04
P30627	Globin‐like protein	glb‐1	CY	0.983	0.594	6.65E‐04	1.90E‐02
P91253	Probable glutathione S‐transferase 7	gst‐7	Unclassified	0.953	1.391	7.19E‐03	3.94E‐04
P34183	RNA ligase	hrs‐1	CY. M	0.867	0.965	2.32E‐03	1.32E‐03
P90732	C‐type LECtin	clec‐41	Unclassified	0.786	0.768	2.46E‐03	3.07E‐03
Q18040	Probable ornithine aminotransferase, mitochondrial	C16A3.10	M	0.784	1.059	5.95E‐03	3.38E‐04
Q23604	FIP (Fungus‐Induced Protein) Related	CELE_ZK813.3	Secr	0.759	1.925	1.51E‐03	4.17E‐05
P91020	Aldo_ket_red domain‐containing protein C07D8.6	C07D8.6	CY	0.605	0.703	1.39E‐03	1.30E‐03
Q22235	Endoplasmin homolog	enpl‐1	ER	0.544	0.397	6.65E‐05	2.95E‐03
*Significantly under‐represented proteins shared by both D76N versus ctrl and WT versus ctrl comparisons*							
Q17339	Female germline‐specific tumor suppressor gld‐1	gld‐1	CY, N	−4.025	−3.619	5.91E‐05	9.39E‐06
O16298	ATP synthase subunit epsilon, mitochondrial	hpo‐18	M	−2.625	−3.26	1.22E‐04	1.57E‐04
Q22285	Transthyretin‐like protein 46	ttr‐46	Secr	−2.17	−2.372	1.93E‐03	1.61E‐02
C6KRN1	Suppressor of aph‐1	sao‐1	CY	−2.086	−2.304	3.02E‐04	1.40E‐04
H2KZA3	ZM domain‐containing protein	pqn‐22	Unclassified	−1.967	−1.581	6.42E‐04	4.63E‐02
O45391	Gamma‐cystathionase	cth‐1	CY	−1.918	−1.652	3.65E‐03	7.96E‐05
P34455	Probable aconitate hydratase, mitochondrial	aco‐2	M	−1.839	−0.848	3.60E‐05	3.58E‐03
P19826	Vinculin	deb‐1	CY, Me	−1.792	−1.528	1.31E‐04	4.63E‐02
Q19286	Intermediate filament protein ifb‐2	ifb‐2	CY	−1.726	−1.918	2.67E‐03	1.20E‐03
Q18496	Acetyl‐coenzyme A synthetase	acs‐19	Unclassified	−1.671	−1.641	1.50E‐02	1.92E‐03
Q17334	Alcohol dehydrogenase 1	sodh‐1	CY	−1.667	−0.714	6.09E‐05	4.95E‐03
Q21551	MICOS complex subunit MIC19	chch‐3	M	−1.404	−1.378	6.01E‐03	1.70E‐02
O18693	Fatty Acid CoA Synthetase family	acs‐2	M	−1.363	−1.474	6.37E‐03	2.54E‐03
Q9NEZ8	Enoyl‐CoA Hydratase	ech‐7	M	−1.16	−1.448	2.62E‐05	2.90E‐03
Q95QQ4	5‐aminoimidazole‐4‐carboxamide ribonucleotide formyltransferase	atic‐1	CY	−0.785	−1.589	2.11E‐03	6.93E‐03
O02267	Complex I‐B14.5a	CELE_F45H10.3	M	−0.747	−0.778	3.51E‐03	9.84E‐04
O45599	Chitin‐binding domain protein cbd‐1	cbd‐1	Secr	−0.691	−1.033	3.71E‐03	1.34E‐04
Q95ZS5	Uncharacterized protein CELE_F56A8.3	CELE_F56A8.3	Me	−0.632	−1.498	2.52E‐03	3.98E‐02
A0A6V7QYI0	AQuaPorin or aquaglyceroporin related	aqp‐7	Me	−0.578	−1.301	2.77E‐04	3.61E‐03
*Significantly altered proteins emerging from both WT versus ctrl and D76N versus ctrl comparisons with opposite direction of deregulation*							
P05690	Vitellogenin‐2	vit‐2	Secr	−0.769	0.874	3.42E‐03	2.47E‐03
P06125	Vitellogenin‐5	vit‐5	Secr	−1.301	0.608	1.12E‐03	1.12E‐02

*Note*: log2FC is the logarithm to base 2 of the fold change value, calculated as the ratio between the protein expression levels in transgenic strain versus control. Proteins without a Gene Ontology or Uniprot annotations for cellular location are shown as “Unclassified”.

Abbreviations: CS, cytoskeleton; CY, cytoplasm; ER, endoplasmic reticulum; M, mitochondrion; Me, cell membrane; N, nucleus; R, ribosome; Secr., secreted.

Among the proteins displaying the highest fold change (log2FC >1), hsp‐16.48/hsp‐16.49, ML domain‐containing protein, Hsp‐16.1, lon protease homolog, and isochorismatase domain‐containing protein were overrepresented both in D76N β_2_‐m and in WT β_2_‐m worms (Table 1). Other proteins most overrepresented in D76N β_2_‐m animals included cystathionine gamma‐lyase 2, SHSP domain‐containing protein, and myosin essential light chain. In WT β_2_‐m worms, a twofold amount of these proteins was found, compared to controls: probable glutathione S‐transferase 7, FIP (fungus‐induced protein) related, ornithine aminotransferase, mitochondrial.

Regarding the shared underrepresented proteins, four of them (female germline‐specific tumor suppressor gld‐1, mitochondrial ATP synthase subunit epsilon, transthyretin‐like protein 46, and suppressor of aph‐1) are reduced to at least 25% or less of what observed in controls.

### 3.4 Pathway analysis of proteomic data

Assessment of biological processes GO term enrichment was performed according to STRING pathway analysis, considering all differential proteins in each of the two comparisons (Table 2). “Cellular amino acid metabolic process” was found to be the most statistically relevant enriched term involving differential proteins in both lists. In WT β_2_‐m worms, other significantly involved processes include oxidation–reduction, generation of precursor metabolites and energy, proteolysis and embryo development, whereas germ cell development was enriched in the D76N β_2_‐m strain.

**TABLE 2:** Top enriched Gene Ontology (GO) biological processes and Kyoto Encyclopedia of Genes and Genomes (KEGG) pathways according to STRING pathway analysis of differentially represented proteins in both comparisons WT β_2_‑m vs control and D76N β_2_‑m vs control. Quantitatively increased proteins are highlighted in bold, while decreased proteins are not highlighted. FDR indicate the False Discovery Rate.

D76N vs ctrl		
**GO**	**Proteins**	**FDR**
Cellular amino acid metabolic process	**C16A3.10**, pyr‐1, cth‐1, **cth‐2**, **hars‐1**, **yrs‐2**, acs‐19, acs‐2, ech‐7	0.0248
Germ cell development	atx‐2, cbd‐1, **hars‐1**, gld‐1, gsk‐3	0.0446
**KEGG**	**Proteins**	**FDR**
Fatty acid degradation	acs‐2, sodh‐1, ech‐7	0.0361
Biosynthesis of amino acids	cth‐1, aco‐2, **cth‐2**	0.0452
Butanoate metabolism	**C05C10.3**, ech‐7	0.0458

WT vs ctrl		
**GO**	**Proteins**	**FDR**
Oxidation‐reduction process	B0303.3, **B0334.3**, **rnr‐2**, **C07D8.6**, gpx‐5, art‐1, **dpyd‐1**, sdha‐2, **idh‐2**, **tyr‐4**, **idhb‐1**, **alh‐10**, **ftn‐1**, **ftn‐2**, D2030.4, glrx‐3, **alh‐9**, **hach‐1**, **alh‐8**, **F20D6.11**, ldp‐1, **F23C8.5**, **F27D4.1**, **acdh‐9**, **fasn‐1**, **idhg‐1**, **aagr‐3**, sdhb‐1, isp‐1, **msra‐1**, F45H10.2, F45H10.3, **skpo‐1**, ife‐1, **F53F1.2**, aco‐2, ucr‐1, **idh‐1**, **K08E3.5**, **gpdh‐2**, sodh‐1, **M106.3**, pycr‐1, **aagr‐2**, gpx‐2, **ech‐4**, **cpt‐2**, **kat‐1**, **exc‐15**, **pygl‐1**, **rnr‐1**, **acdh‐7**, ech‐7, dhs‐9, **glrx‐10**, **prdx‐6**, **Y39G8B.1**, nuo‐5, dpy‐18, **Y47G6A.22**, ads‐1, **txl‐1**, Y57G11C.3, Y63D3A.7, alh‐12, **pfk‐1**, **txdc‐17**	2.60E‐22
Generation of precursor metabolites and energy	sdha‐2, **idh‐2**, **idhb‐1**, glrx‐3, **aldo‐2**, dlat‐1, **F23C8.5**, **pyk‐1**, atp‐3, F27D4.1, **idhg‐1**, **aagr‐3**, sdhb‐1, isp‐1, F45H10.2, F45H10.3, aco‐2, **idh‐1**, **K08E3.5**, aagr‐2, **pgk‐1**, **enol‐1**, **pygl‐1**, **glrx‐10**, nuo‐5, Y57G11C.3, Y63D3A.7, **pfk‐1**, **vha‐12**, hpo‐18, **vha‐13**, vha‐19	2.72E‐13
Cellular amino acid metabolic process	**cbl‐1**, **flu‐2**, **C16A3.10**, **dpyd‐1**, **got‐2.1**, **tars‐1**, **srs‐2**, **gst‐42**, **hach‐1**, **alh‐8**, **upb‐1**, cth‐1, **haly‐1**, bcat‐1, cysl‐2, **gmps‐1**, pycr‐1, **grs‐1**, **hars‐1**, **agxt‐1**, **pars‐1**, ctps‐1, gln‐3, wars‐1, cbs‐1, **cth‐2**	3.48E‐11
Proteolysis	rpn‐10, **pbs‐6**, **C05D11.1**, **usp‐14**, **cpr‐6**, **C34B2.6**, **mig‐6** , **cdc‐48.2**, C56G2.7, **pas‐2**, **dpt‐1**, vps‐36, **ufd‐1**, **rpt‐4**, asp‐13, pbs‐7, **F41C3.5**, **F44E7.4**, **skr‐1**, **F48E3.4**, **pam‐1**, ucr‐1, cpr‐9, **dpf‐3**, **pbs‐5**, pbs‐1, **K12C11.1**, ubc‐18, sgt‐1, **dpf‐5**, **pes‐9**, **rpn‐8**, **asp‐4**, **cpl‐1**, asp‐2, **pbs‐4**, ucr‐2.1, **app‐1**, **lap‐2**, **map‐2**, **Y32F6A.5**, spg‐7, **eel‐1**, rpn‐12, rpt‐2, rpt‐5, kin‐19, acs‐19 , cri‐2, epi‐1 , sao‐1, lmtr‐5, **cdk‐1**, **tep‐1**, **tag‐320**, **C30C11.4**, **dnj‐19**, **T14G8.3**, **hsp‐16.11**, **hsp‐16.48**	2.05E‐09
Embryo development ending in birth or egg hatching	cpg‐2, **arf‐1.2**, **rnr‐2**, **mig‐6**, **cdc‐48.2**, **tba‐2**, **uba‐1**, **icd‐1**, lmn‐1, **ufd‐1**, ima‐2, ima‐3, **sip‐1**, **alg‐1**, **pam‐1**, ife‐1, **pat‐10**, **arf‐3**, bcat‐1, **smo‐1**, act‐3, **act‐2**, **cmd‐1**, rsp‐3, phb‐1, dpy‐18, unc‐52, **sar‐1**	7.18E‐08
**KEGG**	**Proteins**	**FDR**
Biosynthesis of amino acids	idh‐2, **idhb‐1**, **got‐2.1**, **sams‐1**, **aldo‐2**, cth‐1, **pyk‐1**, **idhg‐1**, aco‐2, **idh‐1**, bcat‐1, cysl‐2, pycr‐1, **pgk‐1**, **enol‐1**, gln‐3, **pfk‐1**, cbs‐1, **cth‐2**	1.16E‐12
Oxidative phosphorylation	sdha‐2, D2030.4, **vha‐12**, atp‐3, hpo‐18, vha‐5, sdhb‐1, isp‐1, F45H10.2, F45H10.3, F59C6.5, **vha‐15**, ucr‐2.1, nuo‐5, **vha‐13**, vha‐19, Y63D3A.7, cox‐6b, **vha‐9**	6.44E‐10
Proteasome	**rpn‐10**, **pbs‐6**, C56G2.7, **pas‐2**, **rpt‐4**, **rpt‐2**, pbs‐7, **rpt‐5**, **pbs‐5**, pbs‐1, **rpn‐8**, **pbs‐4**, **rpn‐12**	1.91E‐09
Phagosome	**tbb‐2**, mec‐12, **tba‐2**, **vha‐12**, vha‐5, **tba‐4**, **cpl‐1**, act‐3, **act‐2**, **vha‐15**, **dhc‐1**, **vha‐13**, vha‐19, **vha‐9**	7.13E‐09
Glycolysis / gluconeogenesis	**acs‐19, aldo‐2**, **alh‐9**, dlat‐1, **pyk‐1**, **fbp‐1**, sodh‐1, **pgk‐1, enol‐1**, alh‐12, **pfk‐1**	1.05E‐07

Pathway enrichment analysis was also performed by interrogating the KEGG pathway database (Table 2), revealing that “biosynthesis of amino acids” is an enriched pathway in both transgenic strains. The WT β_2_‐m strain showed the highest number of involved metabolic pathways, including oxidation–reduction processes, protein degradation, phagosome activity, and carbohydrate metabolic processes. The significantly affected pathways are reported in Table 2, in which overrepresented proteins are indicated in bold.

### 3.5 Metabolome remodeling in transgenic *C. elegans* strains expressing WT β_2_‐m or D76N β_2_‐m

Metabolomic profile was defined by extracting polar metabolites from samples of transgenic and control worms and performing a combined solution NMR spectroscopy and mass spectrometry analysis. All samples were derived from synchronized populations harvested at day 5 of adulthood (three biological replicates per strain were analyzed, using the same worm batches submitted to proteomic analysis). An untargeted NMR approach was first employed to obtain an overview of the changes at a global scale. For that purpose, unsupervised statistical PCA was applied—prior to assignment—on the matrix of 1D NMR data, allowing to explore clustering patterns of samples.

**FIGURE 5:**
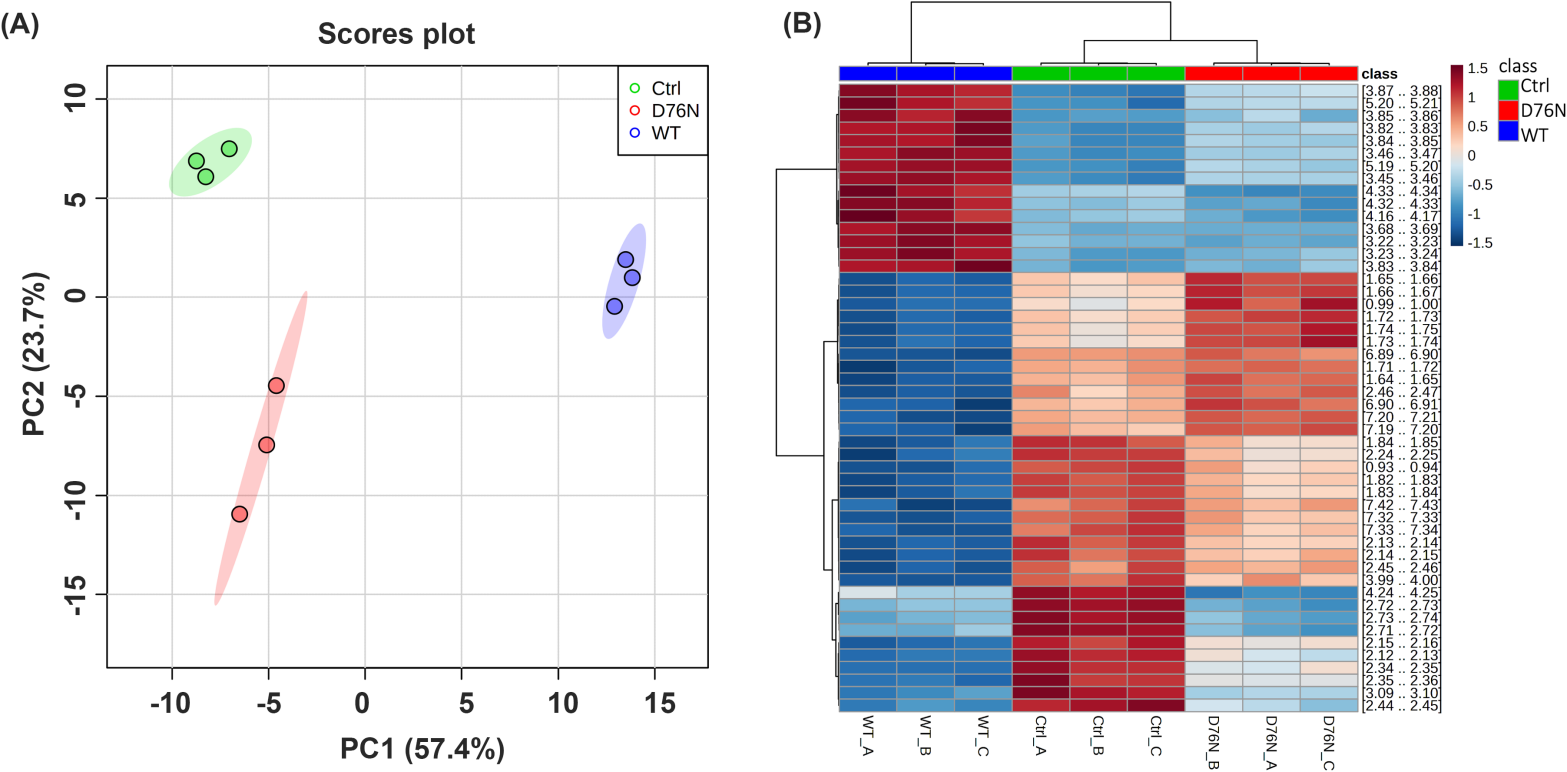
Effects of β_2_‐m isoforms expression on nematode's metabolome. Results from multivariate statistical analysis applied on the 1D‐1H NMR data matrix prior to assignment. (A) PCA for WT β_2_‐m, D76N β_2_‐m and control worms. The explained variances of PC1 and PC2 are shown in brackets. Each point represents one of the replicates of each *C. elegans* strain; 95% confidence regions are displayed. (B) Hierarchical Cluster Analysis Clustering of samples shown with a dendrogram (top of the panel) combined with a heatmap of the top 50 ANOVA variables (1D‐1H NMR buckets' area) emerging from the comparison of β_2_‐m expressing worm strains with control.

A clear discrimination between the three strains was revealed, suggesting that both transgenic lines underwent a significant remodeling of their metabolic profile compared to control worms. Interestingly, WT β_2_‐m worms appeared more altered than D76N worms (Figure 5A) from the control animals. PCA scores plot showed that WT β_2_‐m and control strains were markedly distant from each other along PC1, which accounts for the 57.4% of sample variance. Conversely D76N β_2_‐m worms were not detached from control strain along PC1, but were clustered away from it along PC2, which accounts for sample variance of 23.7%.

Moreover, hierarchical cluster analysis was performed, and results are shown in Figure 5B as a Heatmap, in which the top 50 ANOVA variables (1D‐1H NMR features, that is, window region of the NMR spectra) correspond to rows and the samples to columns. This analysis confirms the significant metabolome remodeling in β_2_‐m worms, which is visibly strain‐specific and only partially overlapping between the two transgenic strains (Figure 5B, top dendrogram).

We then sought to define which metabolites discriminate the β_2_‐m‐expressing worm populations from the control one, and which are specifically altered in each of the two transgenic strains.

The inspection of 1D/2D NMR spectra allowed a partial assignment of NMR signals. A total of 36 metabolites were identified and relatively quantified based on 1D NMR traces. A univariate statistical analysis was performed to compare their levels among the three *C. elegans* strains showing that 21 and 8 metabolic features, mostly belonging to metabolism of amino acids, tricarboxylic acids (TCA), and carbohydrates, were significantly altered for WT β_2_‐m and D76N β_2_‐m worms (Table 3). Among them three shared features were observed namely: glucose‐1P and beta‐glucose that are quantitatively increased and glutamate that is decreased in both transgenic strains in comparison with the control one. Interestingly, the concentrations of trehalose, allantoin, and phosphocholine were higher than control only in the WT β_2_‐m expressing worms. It is worth noting that the last three compounds had been previously associated with stress conditions, aging or misfolding disease in *C. elegans* models.^23^, ^24^, ^25^

**TABLE 3:** NMR‐ identified metabolites varying in WT β_2_‑m and D76N β_2_‑m worms compared to control strain.

*Significantly altered metabolites emerging from WT vs CTR comparison*		
Metabolite	*q*‐value	FC (WT/CTR)
Phenilalanine	0.000052	0.61
Glutamate	0.000052	0.68
Isoleucine	0.000052	0.53
Tyrosine	0.000182	0.70
Glutamine	0.000205	0.59
Valine	0.000205	0.63
Leucine	0.00205	0.60
Lysine	0.000257	0.71
Methionine	0.000456	0.63
Alanine	0.001539	0.73
PhosphoCholine	0.000052	1.57
GlyceroPhosphoCholine	0.000087	1.62
Trehalose	0.000205	1.62
Apha‐Glucose	0.000205	1.31
Glucose‐1P	0.000205	1.93
Aspartate	0.001493	1.58
Betaine	0.001539	1.14
Beta‐Glucose	0.002091	1.50
Allantoin	0.002091	1.31
Lactate/Threonine	0.004626	1.23
Glycine	0.004693	1.33

*Significantly altered metabolites emerging from D76N vs CTR comparison*		
Metabolite	*q*‐value	FC (D76N/CTR)
Inosine	0.000549	0.33
Glutamate	0.003562	0.83
Propionic acid	0.004914	0.64
Glucose‐1P	0.003562	2.29
Beta‐Alanine	0.004914	1.31
Choline	0.006404	1.26
Arginine/beta‐Glucose	0.006771	1.17
Valine	0.009937	1.31

*Note*: Results from multiple groups unpaired *t* test are shown. Only discoveries, i.e. significantly altered metabolites, are reported. Two discoveries have a double label, lactate/threonine and arginine/beta glucose, as the specific NMR peak was compatible with assignments to both compounds. FC indicates the fold change value.

**FIGURE 6:**
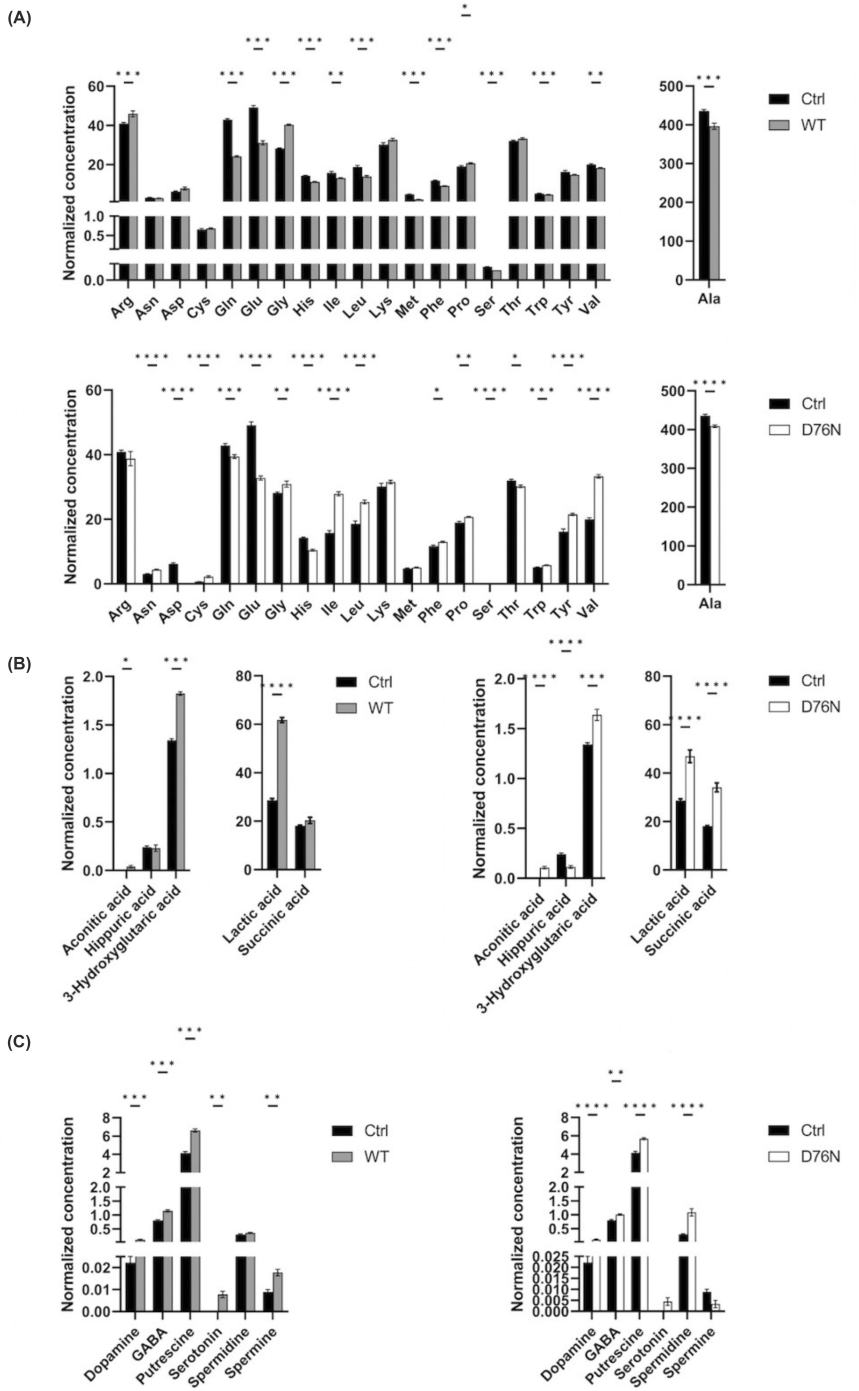
The abundance of (A) amino acids, (B) carboxylic acids, (C) biogenic amines, in WT and D76N β_2_‐m worms in respect to control groups. Plots represent normalized concentrations of metabolites (means ± SEM). The significant differences were evaluated performing Mann–Whitney test for each class of quantified metabolites (*p < 0.05, **p < 0.01, ***p < 0.001, ****p < 0.0001).

A deeper insight into the metabolic profiles was achieved thanks to a targeted MS‐based analysis which allowed covering also additional metabolites besides those already detected as altered by NMR. The assay was indeed able to detect and quantify 62 small molecules (Table S3) including twenty amino acids, nineteen amino acid‐related molecules, one amine oxide, one bile acid, six fatty acids, six biogenic amines, five carboxylic acids, one indoles derivative, two nucleobase‐related metabolite and one cofactor. The “WT β_2_‐m versus ctrl” comparison showed 38 significantly altered metabolites, of which 20 were found overrepresented and 18 underrepresented. A similar number of significant alterations was registered for the “D76N β_2_‐m versus ctrl” comparison: specifically, 23 and 20 molecules were found, respectively, over and underrepresented (Table S3). The profiles of the three most involved metabolite classes, namely amino acids, carboxylic acids, and biogenic amines, are shown in Figure 6. Most of the altered metabolites (39 out of the total 62 identified molecules) belong to the amino acids and amino acid‐related compounds classes (Table S2; Figure 6A). Specifically, six amino acids (Ala, Gln, Glu, Gly, His, Ser) significantly changed by the same direction in both β_2_‐m strains, while five additional ones (Ile, Leu, Phe, Trp, Val) were found decreased or increased in both strains, but with opposite direction of change. Among amino acid‐related compounds, betaine increase is of interest as it plays a role in the *C. elegans* stress response. Another shared altered metabolite was choline, a precursor of betaine, which was increased in both β_2_‐m strains (Table S2).^24^ Regarding carboxylic acids, highest fold changes were found for lactate, succinate, and 3‐hydroxic‐glutaric acid, increased in both transgenics compared to controls. Notably, succinate enters in the TCA cycle, as electron donor to Complex II of the mitochondrial respiratory chain (succinate dehydrogenase) resulting in production of fumarate, while lactate is involved in cellular pathways such as anaerobic glycolysis, gluconeogenesis, and pyruvate metabolism. 3‐hydroxyglutaric acid is a metabolite stemming from glutaric acid and it is mainly described as a product within the catabolic pathway of lysine, tryptohan, and hydroxylysine in humans^26^, ^27^ (Figure 6B). Moreover, a significant increase was observed in transgenic worms for three biogenic amines, namely dopamine, gamma‐aminobutyric acid (GABA), and putresceine (Figure 6C).

It is worth noting that also a number of fatty acids were present in the water‐soluble metabolome of worms and their levels are reduced in D76N β_2_‐m worms (Table S2).

### 3.6 Joint pathway analysis of the proteomic and metabolomic data

**FIGURE 7:**
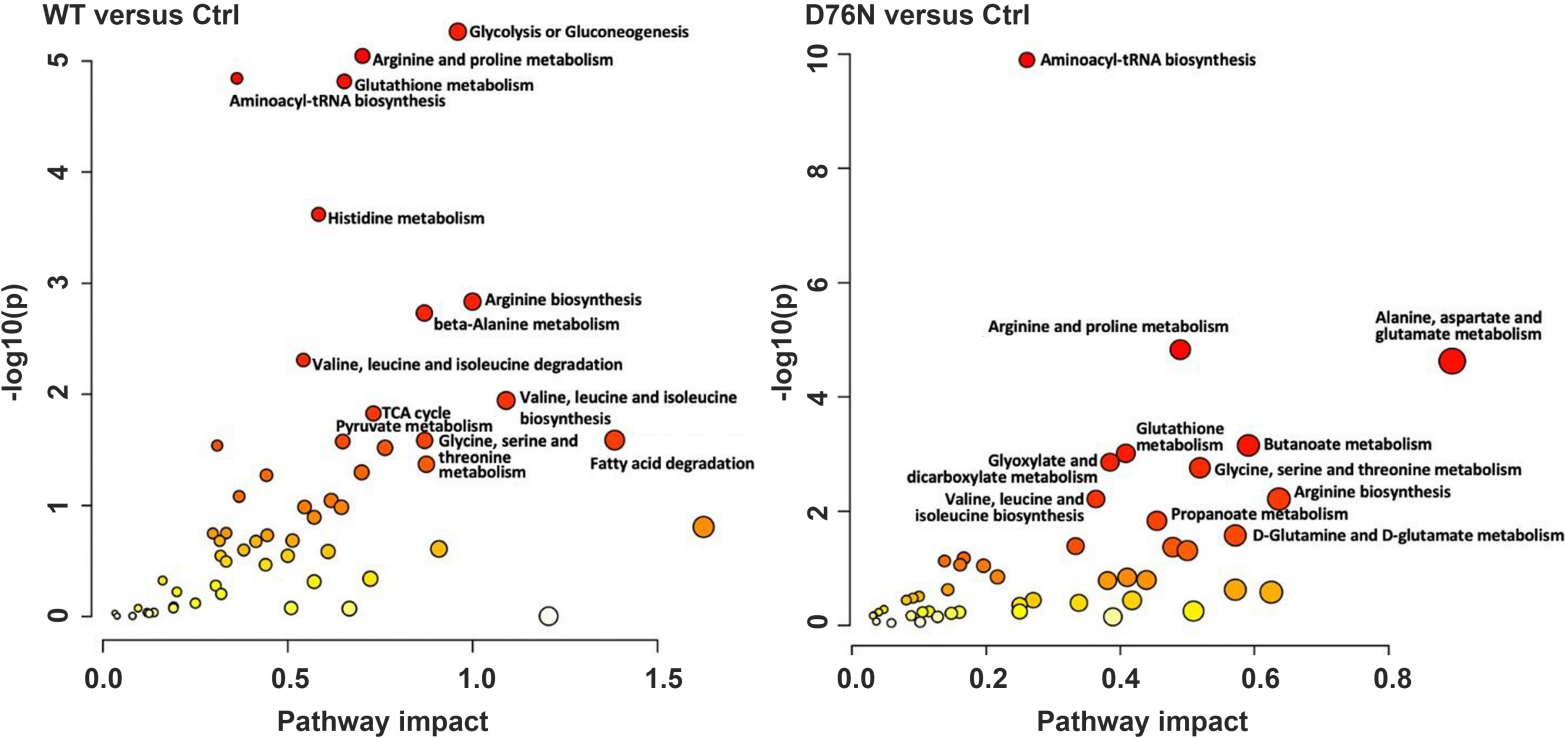
Metabolic pathway enrichment analysis for worms expressing β_2_‐m. Metabolic pathways (nodes) are reported according to the *p*‐values and the impact values; the size of each node is a measure of the number of hits detected within that pathway. For both plots, *p*‐values range from yellow (less significant) to red (more significant).

The interpretation of the multi‐omics results was enhanced by performing an enrichment pathway analysis (Figure 7), combining both the proteomic and the metabolomic (both MS and NMR) data. The statistical significance (*p*‐value) of the pathway enrichment (KEGG *C. elegans* database) was plotted together with “pathway impact,” which measures the incidence of hit proteins and metabolites based on their position within the pathway. A higher number of pathways were found to be significantly involved in WT β_2_‐m rather than in D76N β_2_‐m worms. Specifically, the top‐ranking hit was glycolysis and gluconeogenesis (−log10(*p*) = 5.3115, impact = 0.96) in WT β_2_‐m animals, while arginine and proline metabolism, glutathione metabolism, and aminoacyl‐tRNA biosynthesis were shared in both conditions. Metabolites and proteins significantly involved in each metabolic pathway are reported in Table S3.

## 4 DISCUSSION

In this work we present the thorough phenotypic and molecular characterization of two *C. elegans* transgenic strains expressing distinct isoforms of human β_2_‐m: a newly developed strain producing higher levels of WT β_2_‐m, which mimics pathological conditions such as the ones existing in DRA patients, and a previously described strain expressing at lower levels the D76N β_2_‐m mutant, representative of familial β_2_‐m amyloidosis.

The two engineered strains express β_2_‐m from an integrated array in a smg‐1 mutant that allows transgene induction after upshifting the temperature.^28^ β_2_‐m isoforms are produced in body‐muscle cells with a secretion signal sequence and are present in the worm as unbound, mimicking the pathophysiological conditions occurring in patients, where free β_2_‐m circulates after dissociation from MHCI complex.

Taking advantage of the use of an automated system for the evaluation of worms' survival, larval growth, fertility, and motility^14^, ^20^ we have characterized a pathological phenotype of the WT β_2_‐m‐expressing nematodes, in comparison with the D76N β_2_‐m‐expressing nematodes. Interestingly, both strains show some common features and differences.

A first key finding from this study is that nematodes challenged by higher levels of WT β_2_‐m show an unhealthier phenotype compared to those expressing the highly amyloidogenic D76N β_2_‐m variant in lower amounts. Thus, even though D76N β_2_‐m has lower in vitro stability and higher propensity to aggregate,^7^, ^29^ its proteotoxicity can be overcome in vivo by a higher concentration of WT β_2_‐m. This proteotoxicity is caused by the soluble protein, since we could not detect insoluble aggregates in the nematodes. However, β_2_‐m soluble oligomers were present in both strains and might contribute to damage. We still lack clear evidence on the nature of the toxic species, mainly due to the fact that oligomeric conformers are very transient and instable, and they could be affected by analytical processes employed for their detection.

The fact that the β_2_‐m pre‐fibrillar precursors possess intrinsic toxicity is also concordant with previous experimental evidence on cultured cells.^9^, ^10^, ^11^ It is intriguing to speculate that the toxicity in the worms could be linked to two different key factors known in vitro to subtend distinct forms of β_2_‐m amyloidogenesis: a condition of supersaturation of the partially folded intermediates of WT β_2_‐m, and a condition of partial unfolding due to D76N destabilizing mutation.

The evidence of stress in the two transgenic *C. elegans* strains prompted us to investigate the influence of β_2_‐m expression on the proteome and metabolome of the worm models. Overall, we observed significant remodeling in both strains, which likely results from the combined contribution of β_2_‐m damage to the cells, and of the cells' effort to counteract it. To our knowledge, this is the first global molecular characterization of the derangement in the *C. elegans* proteome and metabolite profile in relation to the expression of a protein causing human systemic amyloidosis. Interestingly, the two approaches provided largely concordant results, which strongly point towards specific deranged pathways.

The proteome changes, especially in the WT β_2_‐m strain, are pronounced and diversified, and significantly involve key biological functions. This remodeling, however, is not fully coincident in the two transgenics. Understanding the changes in their fullness is complex, and the precise role of some specific differential proteins will likely be clarified only through further focused studies.

A first important hint from the proteomic data is that expression of both β_2_‐m isoforms is a significant challenge for the proteostasis system and prompts the worm to increase the production of chaperone proteins in all subcellular compartments. The most prominently shared upregulated proteins, in fact, include cytosolic chaperonines (hsp 16.48, hsp 16.1, hsp 17), as well as species involved in folding within the endoplasmic reticulum (endoplasmin) and mitochondrion (lon protease homolog). This remodeling is especially pronounced in the WT β_2_‐m worms, in which the amount of β_2_‐m is higher. In this strain, tens of proteins involved in protein quality control (as also reflected by the “proteolysis” and “proteasome” enriched term in the pathways analysis) were affected, the vast majority of which are overrepresented. Given that β_2_‐m is eventually secreted and that protein misfolding and amyloidogenesis in systemic amyloidoses are extracellular events, we then sought to investigate if proteins involved in extracellular proteostasis were also quantitatively affected. Gallotta and colleagues^30^ recently reported 57 extracellular regulators of protein aggregation. Among them we identified 4 differential proteins in our β_2_‐m strains: cmd‐1 and ule‐1 are up represented in WT strain while clec‐41 is up represented in both WT and D76N animals. Clec‐1 instead is reduced in WT β_2_‐m nematodes.

A second consideration concerns the important metabolic remodeling, which is evident both from the variation in enzymes involved in metabolic reactions, biosynthesis and processing of energy substrates, as well as from the changes in specific metabolites. The metabolic impact of protein misfolding diseases had been potently suggested, in several past studies, by increasing evidence that pointed to mitochondria as key targets of damage. We now provide a molecular description of these changes in the worm. Mitochondrial enzymes involved in oxidative phosphorylation are concordantly reduced in both strains (e.g., ATP synthase subunit epsilon and Complex I), and especially in WT β_2_‐m worms. Together with the decrease of proteins involved in fatty acids degradation, especially evident in the D76N strain, and with the remodeling of Krebs cycle, the impairment in oxidative phosphorylation may suggest that the mitochondrial aerobic metabolism of the worm is affected, and that the usage of energetic substrates is shifted toward other pathways, such as glycolysis (e.g., concomitant increase of glycolytic enzymes, especially in WT β_2_‐m worms, such as pgk‐1, enol‐1, aldo‐2, pyk‐1). This is in line with metabolomics results, showing that the concentrations of lactate were increased in transgenic worms. Moreover β_2_‐m expressing nematodes present higher levels of glucose‐1‐phosphate than control, suggesting a possible metabolic imbalance of carbon storage.^31^, ^32^, ^33^ As a matter of fact, phosphorylase pygl‐1, the enzyme responsible for phosphorolytic cleavage of glycogen to produce glucose‐1‐phosphate, was found overexpressed in β_2_‐m worms according to proteomic analysis.

Regarding aforementioned sugar storage, we found a relevant trehalose level increment in WT β_2_‐m‐expressing nematodes. Besides its role in carbohydrate storage and transport, trehalose also acts as a cytoprotectant against cold, heat, dehydration, hypoxic, and oxidative insult in invertebrates, most likely by stabilizing the proteome and lipid membranes.^34^ It is also known to increase in aged worms^23^ and may act as an autophagy activator, promoting the clearance of aggregate‐prone proteins.^35^ In addition, research on the long‐lived insulin/IGF‐1‐like signaling mutants of *C. elegans* suggests that enhanced proteostasis is obtained by stabilizing the proteome through increased level of protectants such as trehalose.^34^ The observed increase in trehalose in our worms provides further support to the aforementioned stimulation of proteostasis in these transgenic animals.

WT β_2_‐m‐expressing nematodes let us observe also another interesting feature known to be connected to stress in *C. elegans*. Allantoin, which is the product of a nonenzymatic oxidation reaction when uric acid is exposed to reactive oxygen species (ROS), was found to be overrepresented in WT β_2_‐m. Allantoin was reported to be upregulated in Aβ expressing *C. elegans* strains,^25^ matching with previous observations in AD mice models^36^ Moreover, it has been proposed as a marker of oxidative stress in humans.^37^, ^38^

Another hallmark event common to both transgenic strains was the quantitative alteration of specific amino acids and of proteins involved in amino acids metabolism. Indeed, the level of several amino acids was decreased in transgenic worms; it is intriguing to speculate that readily available amino acids could be used to replenish the TCA cycle either by forming cycle intermediates (glucogenic pathway) or acetylCoA (ketogenic pathway). It is worth noting that an amino acid imbalance is known to activate different signaling pathways in the worm, including some involved in aging and age‐related diseases.^39^, ^40^ Indeed, levels of most amino acids are known to decrease with aging in *C. elegans*.^39^, ^41^ Two of the amino acids that were instead overrepresented in our nematodes are glycine and aspartate. These amino acids had been reported to increase in the worm with aging,^41^, ^42^ although there is no complete accordance on this point, since other studies only agree on the increase in glycine.^39^ Overall, our data prompted us to the attractive speculation that amyloidogenic proteins expression might accelerate the aging process and determine early metabolic alterations. Importantly, our observation of amino acid decrease has a counterpart in amyloid disease in humans, as reported in a study by van der Velpen and colleagues^43^ in which they showed that the glucogenic and ketogenic amino acids, producing intermediates that feed into the TCA cycle, had lower concentrations in patients with Alzheimer disease in both plasma and cerebrospinal fluid compared to controls.

Decreased amino acids were also found in systemic hereditary transthyretin amyloidosis patients^44^ in whom branched‐chain amino acids were underrepresented, possibly reflecting their potential use as source of energy due to stress conditions.

A separate mention is deserved by glutamate, whose reduction in our transgenic worms is paralleled by an increased abundance of gcs‐1 (Q20117 glutamate‐cysteine ligase) in WT β_2_‐m nematodes. This protein catalyzes glutathione synthesis from Glutamate and Cysteine, suggesting a high consumption of the former. Although we could not identify glutathione by NMR, we may speculate that nematodes try to increase its synthesis to counteract oxidative stress caused by the presence of β_2_‐m. Additional differential proteins identified by proteomic analysis (in particular gst‐4, gst‐7, gpx‐2, prdx‐6, C32D5.8) are enzymes involved in detoxification and in regulation of the cellular redox state.^45^ These findings are consistent with the increase in ROS levels in β_2_‐m‐expressing worms and with the aforementioned metabolomics data. Interestingly, oxidative stress and impaired redox balance, probably related to mitochondrial dysfunction, are indeed a well‐known early event occurring in vivo and in experimental models of neurodegeneration, as well as of systemic amyloidosis. In a previously characterized *C. elegans* model of immunoglobulin light chains proteotoxicity, significant mitochondrial redox stress was demonstrated to be specifically associated with exposure to amyloidogenic light chains from patients with light chain amyloidosis.^46^

Overall, according to our results, it could be speculated that the exposure to the proteotoxic stress embodied by the intrinsically misfolding‐prone β_2_‐m, poses a threat for the correct maintenance of the whole organism proteostasis, accelerating the functional decline of tissues. It is worth noting that a reduction in the ability of proteostasis network to protect cells from protein instability has been observed in *C. elegans* in the context of normal aging.^47^ Moreover, this first characterization of proteomic and metabolomic alterations linked to the expression in *C. elegans* of proteins responsible for systemic amyloidosis in humans, highlights the relevance of “omics” approaches for identifying potential biomarkers of amyloid disease in the *C. elegans* model and, therefore, providing further clues to understand the molecular basis of amyloid cytotoxicity.

## AUTHOR CONTRIBUTIONS

V.B., S.R., and S.G., conceived the experiments. S.R., G.F., V.M., L.M., P.N., MC. M., A.B., D.C., and M.C., conducted the experiments and analyzed the results. S.R., S.G., F.L., PP. M., V.B., G.V., and M.R. wrote and discussed the manuscript. All authors reviewed and approved the manuscript.

## CONFLICT OF INTEREST STATEMENT

The authors declare no conflicts of interest.

## Supporting information


Data S1:

## Data Availability

The mass spectrometry proteomics data that support the findings of this study, are openly available at the ProteomeXchange Consortium (http://www.proteomexchange.org) via the PRIDE partner repository with the dataset identifier PXD04023. Reviewer account details: username: reviewer_pxd040230@ebi.ac.uk Password: YMl10EDj. Any other data supporting this study are available in the methods, results and/or supplementary material of this article.

## References

[1] Stoppini M , Bellotti V . Systemic amyloidosis: lessons from β2‐microglobulin. J Biol Chem. 2015;290:9951‐9958. 25750126 10.1074/jbc.R115.639799 PMC4400370

[2] Wieczorek M , Abualrous ET , Sticht J , et al. Major histocompatibility complex (MHC) class I and MHC class II proteins: conformational plasticity in antigen presentation. Front Immunol. 2017;8:292. 28367149 10.3389/fimmu.2017.00292 PMC5355494

[3] Floege J , Bartsch A , Schulze M , Shaldon S , Koch KM , Smeby LC . Clearance and synthesis rates of beta 2‐microglobulin in patients undergoing hemodialysis and in normal subjects. J Lab Clin Med. 1991;118:153‐165. 1856578

[4] Zumrutdal A . Role of β2‐microglobulin in uremic patients may be greater than originally suspected. World J Nephrol. 2015;4:98‐104. 25664251 10.5527/wjn.v4.i1.98 PMC4317633

[5] Althubiti M , Elzubier M , Alotaibi GS , et al. Beta 2 microglobulin correlates with oxidative stress in elderly. Exp Gerontol. 2021;150:111359. 33905876 10.1016/j.exger.2021.111359

[6] Valleix S , Gillmore JD , Bridoux F , et al. Hereditary systemic amyloidosis due to Asp76Asn variant β2‐microglobulin. N Engl J Med. 2012;366:2276‐2283. 22693999 10.1056/NEJMoa1201356

[7] Mangione PP , Esposito G , Relini A , et al. Structure, folding dynamics, and amyloidogenesis of D76N β2‐microglobulin: roles of shear flow, hydrophobic surfaces, and α‐crystallin. J Biol Chem. 2013;288:30917‐30930. 24014031 10.1074/jbc.M113.498857 PMC3829406

[8] Mizuno H , Hoshino J , So M , et al. Dialysis‐related amyloidosis associated with a novel β2‐microglobulin variant. Amyloid. 2021;28:42‐49. 32875920 10.1080/13506129.2020.1813097

[9] Giorgetti S , Raimondi S , Cassinelli S , et al. beta2‐microglobulin is potentially neurotoxic, but the blood brain barrier is likely to protect the brain from its toxicity. Nephrol Dial Transplant. 2009;24:1176‐1181. 19008236 10.1093/ndt/gfn623

[10] Leri M , Oropesa‐Nuñez R , Canale C , et al. Oleuropein aglycone: a polyphenol with different targets against amyloid toxicity. Biochim Biophys Acta Gen Subj. 2018;1862:1432‐1442. 29571746 10.1016/j.bbagen.2018.03.023

[11] Goodchild SC , Sheynis T , Thompson R , et al. β2‐microglobulin amyloid fibril‐induced membrane disruption is enhanced by endosomal lipids and acidic pH. PloS One. 2014;9:e104492. 25100247 10.1371/journal.pone.0104492 PMC4123989

[12] Zhang P , Fu X , Sawashita J , et al. Mouse model to study human a beta2M amyloidosis: generation of a transgenic mouse with excessive expression of human beta2‐microglobulin. Amyloid. 2010;17:50‐62. 20462363 10.3109/13506129.2010.483116

[13] Diomede L , Soria C , Romeo M , et al. C. elegans expressing human β2‐microglobulin: a novel model for studying the relationship between the molecular assembly and the toxic phenotype. PloS One. 2012;7:e52314. 23284985 10.1371/journal.pone.0052314 PMC3528749

[14] Faravelli G , Raimondi S , Marchese L , et al. C. elegans expressing D76N β_2_‐microglobulin: a model for in vivo screening of drug candidates targeting amyloidosis. Sci Rep. 2019;9:19960. 31882874 10.1038/s41598-019-56498-5 PMC6934621

[15] Good SC , Dewison KM , Radford SE , van Oosten‐Hawle P . Global proteotoxicity caused by human β2 microglobulin variants impairs the unfolded protein response in c. elegans. Int J Mol Sci. 2021;22:10752. 34639093 10.3390/ijms221910752 PMC8509642

[16] Spaziani S , Imperlini E , Mancini A , Caterino M , Buono P , Orrù S . Insulin‐like growth factor 1 receptor signaling induced by supraphysiological doses of IGF‐1 in human peripheral blood lymphocytes. Proteomics. 2014;14:1623‐1629. 24753496 10.1002/pmic.201300318

[17] Cox J , Hein MY , Luber CA , Paron I , Nagaraj N , Mann M . Accurate proteome‐wide label‐free quantification by delayed normalization and maximal peptide ratio extraction, termed MaxLFQ. Mol Cell Proteomics. 2014;13:2513‐2526. 24942700 10.1074/mcp.M113.031591 PMC4159666

[18] Koyuncu S , Loureiro R , Lee HJ , Wagle P , Krueger M , Vilchez D . Rewiring of the ubiquitinated proteome determines ageing in C. elegans. Nature. 2021;596(7871):285‐290. 34321666 10.1038/s41586-021-03781-z PMC8357631

[19] Caterino M , Costanzo M , Fedele R , et al. The serum metabolome of moderate and severe COVID‐19 patients reflects possible liver alterations involving carbon and nitrogen metabolism. Int J Mol Sci. 2021;22:9548. 34502454 10.3390/ijms22179548 PMC8431319

[20] Partridge FA , Brown AE , Buckingham SD , et al. An automated high‐throughput system for phenotypic screening of chemical libraries on C. Elegans and parasitic nematodes. Int J Parasitol Drugs Drug Resist. 2018;8:8‐21. 29223747 10.1016/j.ijpddr.2017.11.004 PMC5734697

[21] Meratan AA , Ghasemi A , Nemat‐Gorgani M . Membrane integrity and amyloid cytotoxicity: a model study involving mitochondria and lysozyme fibrillation products. J Mol Biol. 2011;409:826‐838. 21565199 10.1016/j.jmb.2011.04.045

[22] Eckert A , Pagani L . Amyloid‐Beta interaction with mitochondria. Int J Alzheimers Dis. 2011;2011:925050. 21461357 10.4061/2011/925050 PMC3065051

[23] Wan QL , Shi X , Liu J , et al. Metabolomic signature associated with reproduction‐regulated aging in Caenorhabditis elegans. Aging. 2017;9:447‐474. 28177875 10.18632/aging.101170 PMC5361674

[24] Trautwein C , MacKinnon N , Korvink JG . Micro‐NMR elucidates altered metabolites in the Parkinson's disease‐related catp‐6 genotype of Caenorhabditis elegans. Metabolomics. 2017;4:1‐10.

[25] Van Assche R , Temmerman L , Dias DA , et al. Metabolic profiling of a transgenic Caenorhabditis elegans Alzheimer model. Metabolomics. 2015;11:477‐486. 25750603 10.1007/s11306-014-0711-5 PMC4342517

[26] Peters V , Morath M , Mack M , et al. Formation of 3‐hydroxyglutaric acid in glutaric aciduria type I: in vitro participation of medium chain acyl‐CoA dehydrogenase. 2019. 10.1002/jmd2.12026 PMC6498835 31240164

[27] Leandro, J. and Houten, S. M. (2020) The lysine degradation pathway: subcellular compartmentalization and enzyme deficiencies. 10.1016/j.ymgme.2020.07.010 32768327

[28] Mango SE . Stop making nonSense: the C. Elegans smg genes. Trends Genet. 2001;17:646‐653. 11672865 10.1016/s0168-9525(01)02479-9

[29] Natalello A , Mangione PP , Giorgetti S , et al. Co‐fibrillogenesis of wild‐type and D76N β2‐microglobulin: THE CRUCIAL ROLE OF FIBRILLAR SEEDS*. J Biol Chem. 2016;291:9678‐9689. 26921323 10.1074/jbc.M116.720573 PMC4850305

[30] Gallotta I , Sandhu A , Peters M , et al. Extracellular proteostasis prevents aggregation during pathogenic attack. Nature. 2020;584:410‐414. 32641833 10.1038/s41586-020-2461-z

[31] Seo Y , Kingsley S , Walker G , Mondoux MA , Tissenbaum HA . Metabolic shift from glycogen to trehalose promotes lifespan and healthspan in Caenorhabditis elegans. Proc Natl Acad Sci U S A. 2018;115:E2791‐E2800. 29511104 10.1073/pnas.1714178115 PMC5866546

[32] Rasulova M , Zečić A , Moreno JMM , Vandemeulebroucke L , Dhondt I , Braeckman BP . Elevated Trehalose levels in C. Elegans daf‐2 mutants increase stress resistance, not lifespan. Metabolites. 2021;11:1‐14. 10.3390/metabo11020105 PMC7917784 33673074

[33] Zečić A , Braeckman BP . DAF‐16/FoxO in Caenorhabditis elegans and its role in metabolic remodeling. Cell. 2020;9:109. 10.3390/cells9010109 PMC7017163 31906434

[34] Depuydt G , Shanmugam N , Rasulova M , Dhondt I , Braeckman BP . Increased protein stability and decreased protein turnover in the Caenorhabditis elegans ins/IGF‐1 daf‐2 mutant. J Gerontol A Biol Sci Med Sci. 2016;71:1553‐1559. 26865495 10.1093/gerona/glv221 PMC5106850

[35] Sarkar S , Davies JE , Huang Z , Tunnacliffe A , Rubinsztein DC . Trehalose, a novel mTOR‐independent autophagy enhancer, accelerates the clearance of mutant huntingtin and alpha‐synuclein. J Biol Chem. 2007;282:5641‐5652. 17182613 10.1074/jbc.M609532200

[36] Fukuhara K , Ohno A , Ota Y , et al. NMR‐based metabolomics of urine in a mouse model of Alzheimer's disease: identification of oxidative stress biomarkers. J Clin Biochem Nutr. 2013;52:133‐138. 23526113 10.3164/jcbn.12-118 PMC3593130

[37] Yardim‐Akaydin S , Sepici A , Özkan Y , Şimşek B , Sepici V . Evaluation of allantoin levels as a new marker of oxidative stress in Behçet's disease. Scand J Rheumatol. 2006;35:61‐64. 16467045 10.1080/03009740510026878

[38] Kand'ár R , Žáková P . Allantoin as a marker of oxidative stress in human erythrocytes. Clin Chem Lab Med. 2008;46:1270‐1274. 18636793 10.1515/CCLM.2008.244

[39] Canfield CA , Bradshaw PC . Amino acids in the regulation of aging and aging‐related diseases. Transl Med Aging. 2019;3:70‐89.

[40] Edwards C , Canfield J , Copes N , et al. Mechanisms of amino acid‐mediated lifespan extension in Caenorhabditis elegans. BMC Genet. 2015;16:8. 25643626 10.1186/s12863-015-0167-2 PMC4328591

[41] Hastings J , Mains A , Virk B , et al. Multi‐omics and genome‐scale modeling reveal a metabolic shift during C. elegans aging. Front Mol Biosci. 2019;6:2. 30788345 10.3389/fmolb.2019.00002 PMC6372924

[42] Liu YJ , Janssens GE , McIntyre RL , et al. Glycine promotes longevity in Caenorhabditis elegans in a methionine cycle‐dependent fashion. PLoS Genet. 2019;15:e1007633. 30845140 10.1371/journal.pgen.1007633 PMC6424468

[43] van der Velpen V , Teav T , Gallart‐Ayala H , et al. Systemic and central nervous system metabolic alterations in Alzheimer's disease. Alzheimers Res Ther. 2019;11:93. 31779690 10.1186/s13195-019-0551-7 PMC6883620

[44] Olsson M , Hellman U , Wixner J , Anan I . Metabolomics analysis for diagnosis and biomarker discovery of transthyretin amyloidosis. Amyloid. 2021;28:234‐242. 34319177 10.1080/13506129.2021.1958775

[45] Ferguson GD , Bridge WJ . The glutathione system and the related thiol network in Caenorhabditis elegans. Redox Biol. 2019;24:101171. 30901603 10.1016/j.redox.2019.101171 PMC6429583

[46] Diomede L , Rognoni P , Lavatelli F , et al. A Caenorhabditis elegans‐based assay recognizes immunoglobulin light chains causing heart amyloidosis. Blood. 2014;123:3543‐3552. 24665135 10.1182/blood-2013-10-525634 PMC4047494

[47] Huang C , Wagner‐Valladolid S , Stephens AD , et al. Intrinsically aggregation‐prone proteins form amyloid‐like aggregates and contribute to tissue aging in *Caenorhabditis elegans* . Elife. 2019;8:e43059. 31050339 10.7554/eLife.43059 PMC6524967

