# Supplementary material for: Human wild‐type and D76N β2‐microglobulin variants are significant proteotoxic and metabolic stressors for transgenic C. elegans
Source: FASEB Bioadv. 2023 Oct 25;5(11):484–505. doi: 10.1096/fba.2023-00073 (PMC10626158; doi:10.1096/fba.2023-00073)
Supplement: Supplementary file 1 — Data S1: [file FBA2-5-484-s001.pdf]

Supplementary information

NMR acquisition and processing details

1D 1H NMR spectra was acquired with water peak suppression and a standard NOESY (1) pulse sequence (Bruker pulse program *noesypr1d*) using 32 scans, 65536 data points, a spectral width of 13888.9 Hz, an acquisition time of 2.7 s, a relaxation delay of 10 s and a mixing time of 0.1 s.

The 1D NMR spectra were processed and analyzed with the software ACD-Labs Spectrus Processor 2020.2.0. After FID apodization with an exponential multiplication window function (LB 0.3), all spectra were phased, Fourier-transformed and chemical-shift referenced.

A set of two-dimensional NMR spectra was also acquired for each sample. In particular, homonuclear 2D 1H-1H-NMR experiments included correlation spectroscopy (COSY) and total correlation spectroscopy (TOCSY) along with heteronuclear 1H, 13C single quantum coherence (1H-13C-HSQC) (2).

Gradient enhanced magnitude COSY pulse sequence (*cosygpprqf* supplied by Bruker) with a presaturation during relaxation delay was used. Spectra were collected with 2048 points in t2 and 256 points in t1 over a sweep width of 9615.385 Hz, with 8 scans per t1 value. The acquisition times were fixed to 0.11 s in F2 and 0.027s in F1, the relaxation delay to 2 s. The TOCSY pulse sequence (*mlevphpr.2* supplied by Bruker) included: coherence transfer during a multiple-pulse spin-lock period, solvent suppression by presaturation, applied along the 2s relaxation delay, and mixing time of 80 ms. Spectra were collected with 2048 points in t2 and 256 points in t1 over a sweep width of 11363.637 Hz, with 8 scans per t1 value. The acquisition times were fixed to 0.09 s in F2 and 0. 0.01s in F1. The resulting COSY and TOCSY spectra were processed in Topspin 4.1.0 using standard methods, with sine-squared apodization in both dimensions and zero filling in t1 to yield a transformed 2D dataset of 1024 by 1024 points. The 1H, 13C-HSQC pulse sequence (*hsqcetgpsisp2.2* supplied by Bruker) using sensitivity

improvement and shaped-pulses for all 180-degree pulses on 1H – channel, with decoupling during acquisition. Spectra were collected with 2048 points in t2 and 128 points in t1 over a sweep width of 9615.385 Hz for 1H and 31691.145 Hz for 13C, with 16 scans per t1 value. The acquisition times were fixed to 0.11s in F2 and 0.002s in F1, the relaxation delay to 2 s. The resulting HSQC spectra were processed in Topspin 4.1.0 using standard methods, with sine-squared apodization in both dimensions, 0th order phase correction in 1H of 180 degrees and zero filling in t1 to yield a transformed 2D dataset of 1024 by 1024 points.

REFERENCES

1. Mckay, R. T. (2011). How the 1D-NOESY suppresses solvent signal in metabonomics NMR spectroscopy: An examination of the pulse sequence components and evolution. *Concepts in Magnetic Resonance Part A: Bridging Education and Research*, *38 A*(5), 197–220. https://doi.org/10.1002/cmr.a.20223
2. Emwas, A. H., Roy, R., McKay, R. T., Tenori, L., Saccenti, E., Nagana Gowda, G. A., Raftery, D., Alahmari, F., Jaremko, L., Jaremko, M., & Wishart, D. S. (2019). Nmr spectroscopy for metabolomics research. *Metabolites*, *9*(7). https://doi.org/10.3390/metabo9070123

Table S1. Differentially represented proteins in D76N vs ctrl and WT vs ctrl comparisons.
= Significantly altered proteins emerging from both comparisons with the same direction of deregulation; ≠ significantly altered proteins emerging from both comparisons with the same deregulation; CY, cytoplasm; N, nucleus; M, mitochondrion; CS, cytoskeleton; R, ribosome; ER, endoplasmic reticulum; Me, cell membrane; Secr., Secreted. Proteins without a Gene Ontology or Uniprot annotations for cellular location are shown as ‘Unclassified’.

D76N vs ctrl (over-represented proteins, n = 24)						
**Accession**	**Protein**	**Gene**	**Localization**	**log2FC**	**p-value**	**Notes**
A0A0K3ASH9	PAW domain-containing protein	CELE_Y50D4B.4	CY. N	2.976	0.000986	
O62415	Lysozyme-like protein 1	lys-1	CY	1.767	0.0128	
P02513	Heat shock protein Hsp-16.48/Hsp-16.49	hsp-16.48	Unclassified	1.73	0.00352	=
Q20311	ML domain-containing protein	CELE_F42A10.6	Unclassified	1.665	0.0164	=
P34696	Heat shock protein Hsp-16.1/Hsp-16.11	hsp-16.1	Unclassified	1.496	0.0000878	=
O44952	Lon protease homolog. mitochondrial	C34B2.6	M	1.379	0.00852	=
P55216	Putative cystathionine gamma-lyase 2	cth-2	CY	1.376	0.00634	=
Q20660	SHSP domain-containing protein	hsp-17	Unclassified	1.327	0.000682	=
Q20062	Isochorismatase domain-containing protein	marb-1	CY	1.243	0.0123	=
G5ED95	Tyrosine--tRNA ligase	yrs-2	Unclassified	1.158	0.000849	
P53014	Myosin. essential light chain	mlc-3	CY	1.151	0.000105	=
H2KZV5	Lipase_3 domain-containing protein	CELE_T21H3.1	Unclassified	1.08	0.000501	
P30627	Globin-like protein	glb-1	CY	0.983	0.000665	=
P91253	Probable glutathione S-transferase 7	gst-7	Unclassified	0.953	0.00719	=
O45444	C-type LECtin clec-63	clec-63	Unclassified	0.947	0.00366	
O01530	Aspartic protease 6	asp-6	Secr	0.92	0.000209	
P34183	RNA ligase	hars-1	CY. M	0.867	0.00232	=
O17365	SERPIN domain-containing protein	srp-2	Secr	0.856	0.00688	
P90732	C-type LECtin	clec-41	Unclassified	0.786	0.00246	=
Q18040	Probable ornithine aminotransferase, mitochondrial	C16A3.10	M	0.784	0.00595	=
Q23604	FIP (Fungus-Induced Protein) Related	CELE_ZK813.3	Secr	0.759	0.00151	=
Q09450	Probable succinyl-CoA:3-ketoacid coenzyme A transferase, mitochondrial	C05C10.3	M	0.667	0.00114	
P91020	Aldo_ket_red domain-containing protein C07D8.6	C07D8.6	CY	0.605	0.00139	=
Q22235	Endoplasmin homolog	enpl-1	ER	0.544	0.0000665	=
D76N vs ctrl (under-represented proteins, n = 38)						
**Accession**	**Protein**	**Gene**	**Localization**	**log2FC**	**p-value**	**Notes**
O17646	USP domain-containing protein	C34F6.9	Unclassified	-5.416	0.000197	
Q17339	Female germline-specific tumor suppressor gld-1	gld-1	CY, N	-4.025	0.0000591	=
Q18994	eIF-2B GDP-GTP exchange factor subunit epsilon	eif-2bepsilon	CY	-3.121	0.0000396	

O61977	TransThyretin-Related family domain ttr-47	ttr-47	Secr	-2.735	0.0184	
O16298	ATP synthase subunit epsilon, mitochondrial	hpo-18	M	-2.625	0.000122	=
Q18074	Uncharacterized protein C18B2.3	C18B2.3	Unclassified	-2.375	0.0229	
Q02328	Huntington interacting protein related 1	hipr-1	CY	-2.188	0.00598	
Q22285	Transthyretin-like protein 46	ttr-46	Secr	-2.17	0.00193	=
C6KRN1	Suppressor of aph-1	sao-1	CY	-2.086	0.000302	=
H2KZA3	ZM domain-containing protein	pqn-22	Unclassified	-1.967	0.000642	=
O45391	Gamma-cystathionase	cth-1	CY	-1.918	0.00365	=
Q9U2Q9	Glycogen synthase kinase-3	gsk-3	CY, N	-1.908	0.00966	
Q18990	CAD protein	pyr-1	CY	-1.865	0.0015	
P34455	Probable aconitate hydratase, mitochondrial	aco-2	M	-1.839	0.000036	=
G5ED29	Ataxin-2 homolog	atx-2	CY, N	-1.792	0.00377	
P19826	Vinculin	deb-1	CY, Me	-1.792	0.000131	=
O45518	Arginine kinase	argk-1	Unclassified	-1.727	0.009	
Q19286	Intermediate filament protein ifb-2	ifb-2	CY	-1.726	0.00267	=
Q18496	Acetyl-coenzyme A synthetase	acs-19	Unclassified	-1.671	0.015	=
Q17334	Alcohol dehydrogenase 1	sodh-1	CY	-1.667	0.0000609	=
Q21551	MICOS complex subunit MIC19	chch-3	M	-1.404	0.00601	=
O18693	Fatty Acid CoA Synthetase family	acs-2	M	-1.363	0.00637	=
P06125	Vitellogenin-5	vit-5	Secr	-1.301	0.00112	≠
Q19766	Mitochondrial import receptor subunit TOM20 homolog	tomm-20	M	-1.289	0.00471	
P55155	Vitellogenin-1	vit-1	Secr	-1.264	0.000144	
Q9NEZ8	Enoyl-CoA Hydratase	ech-7	M	-1.16	0.0000262	=
P18947	Vitellogenin-4	vit-4	Secr	-1.125	0.00787	
Q19289	Intermediate filament protein ifb-1	ifb-1	CY	-0.87	0.0046	
Q19869	60S ribosomal protein L26	rpl-26	R	-0.854	0.00562	
O02485	Uncharacterized protein ZK1073.1	ZK1073.1	Unclassified	-0.804	0.00245	
Q95QQ4	5-aminoimidazole-4-carboxamide ribonucleotide formyltransferase	atic-1	CY	-0.785	0.00211	=
P05690	Vitellogenin-2	vit-2	Secr	-0.769	0.00342	≠
P90901	Intermediate filament protein ifa-1	ifa-1	CY	-0.754	0.00252	
O02267	Complex I-B14.5a	CELE_F45H10.3	M	-0.747	0.00351	=
O45599	Chitin-binding domain protein cbd-1	cbd-1	Secr	-0.691	0.00371	=
Q95ZS5	Leucine-rich repeat domain, L domain-like	CELE_F56A8.3	Me	-0.632	0.00252	=
Q8WQA8	40S ribosomal protein S20	rps-20	R	-0.619	0.00322	
A0A6V7QYI0	AQuaPorin or aquaglyceroporin related	aqp-7	Me	-0.578	0.000277	=
WT vs ctrl (over-represented proteins, n = 300)						
**Accession**	**Protein**	**Gene**	**Localization**	**log2FC**	**p-value**	**Notes**
O44730	Zwei Ig domain protein zig-7	zig-7	Secr	3.172	0.005	

Q20173	TWinFilin actin binding protein homolog	twf-2	CY	2.937	0.000359	
O16453	Ferritin	ftn-1	CY	2.901	0.00792	
Q95Y29	Nematode Specific Peptide family	CELE_Y34B4A.6	Unclassified	2.658	0.00357	
G5EDP0	Retinoid-inducible serine carboxypeptidase	CELE_Y32F6A.5	Unclassified	2.635	0.0267	
O01780	Uterine Lumin Expressed/locailized	ule-1	Secr	2.593	0.036	
O17919	Putative H/ACA ribonucleoprotein complex subunit 4	K01G5.5	N	2.473	0.0465	
Q18822	Aldedh domain-containing protein alh-10	alh-10	Unclassified	2.462	0.00353	
O17893	Secreted protein	CELE_F55B11.3	Unclassified	2.244	0.00168	
O17345	TransThyretin-Related family domain ttr-6	ttr-6	Secr	2.234	0.0116	
O62289	TransThyretin-Related family domain ttr-51	ttr-51	Secr	2.12	0.00106	
P34500	Transthyretin-like protein 2	ttr-2	Secr	2.059	0.000297	
G5EEV5	5C820	pud-3	Unclassified	1.957	0.0000775	
Q9U2F6	Related to yeast Vacuolar Protein Sorting factor	vps-2	CY	1.938	0.0231	
O02089	Peptide-methionine (S)-S-oxide reductase	msra-1	CY	1.938	0.00409	
Q23604	Secreted protein	CELE_ZK813.3	Secr	1.925	0.0000417	=
O44144	PERMeable eggshell perm-4	perm-4	Unclassified	1.838	0.00107	
Q10943	ADP-ribosylation factor 1-like 2	arf-1.2	CY	1.82	0.032	
Q19063	Cuticle protein	CELE_E04F6.8	Unclassified	1.794	0.00137	
Q19191	Uncharacterized protein F08B12.4	F08B12.4	N	1.714	0.00205	
Q9GUF2	ACid Phosphatase family	acp-6	Unclassified	1.675	0.000152	
P34383	Fatty-acid and retinol-binding protein 2	far-2	Secr	1.652	0.012	
Q9U1X9	60S acidic ribosomal protein P2	rla-2	CY	1.574	0.000478	
Q18947	Uterine Lumin Expressed/locailized ule-3	ule-3	Unclassified	1.565	0.00203	
Q18594	Uncharacterized protein C44B7.5	C44B7.5	Secr	1.563	0.00272	
P02513	Heat shock protein Hsp-16.48/Hsp-16.49	hsp-16.48	Unclassified	1.553	0.00228	=
Q11174	Probable endochitinase	cht-1	Secr	1.545	0.00295	
O01497	TYRosinase	tyr-4	Unclassified	1.537	0.0112	
Q20224	Fatty acid-binding protein homolog 2	lbp-2	Secr	1.535	0.000714	
O45346	Notch ligand osm-11	osm-11	Me	1.481	0.0067	
P10984	Actin-2	act-2	CY	1.481	0.0337	
G4SF79	Ig-like domain-containing protein	CELE_F25E2.2	Secr	1.471	0.000179	
Q2EEM8	TransThyretin-Related family domain ttr-45	ttr-45	Secr	1.452	0.00496	
Q21265	Putative metalloproteinase inhibitor tag-225	tag-225	Secr	1.451	0.00801	
O17389	Thymosin beta	tth-1	CY	1.45	0.00214	
Q18529	Peritrophic matrix protein	C39D10.7	Secr	1.434	0.000133	
G5EC22	CUB_2 domain-containing protein CELE_F55G11.4	CELE_F55G11.4	Unclassified	1.406	0.0000368	

P91253	Probable glutathione S-transferase 7	gst-7	Unclassified	1.391	0.000394	=
G5EC10	Galectin lec-9	lec-9	CY	1.385	0.0316	
Q9GPA1	Peptidase S1 domain-containing protein	CELE_F48E3.4	Unclassified	1.362	0.000104	
P34696	Heat shock protein Hsp-16.1/Hsp-16.11	hsp-16.1	Unclassified	1.358	0.00354	=
Q94051	Caveolin-1	cav-1	CY, Me	1.35	0.00685	
Q20616	Peroxidase skpo-1	skpo-1	Unclassified	1.329	0.000926	
Q7YTU0	TEP (ThiolEster containing Protein)	tep-1	Secr	1.328	0.00272	
Q9N456	Glutaredoxin domain-containing protein glrx-10	glrx-10	M	1.294	0.0221	
P90889	Uncharacterized protein CELE_F55H12.4	CELE_F55H12.4	Unclassified	1.273	0.0292	
O02115	Proliferating cell nuclear antigen	pcn-1	N	1.269	0.000879	
Q20311	ML domain-containing protein	CELE_F42A10.6	Unclassified	1.268	0.0321	=
O16264	Phosphatidylethanolamine-binding protein homolog F40A3.3	F40A3.3	M	1.248	0.0255	
Q20062	Isochorismatase domain-containing protein	marb-1	CY	1.246	0.000639	=
Q18143	Chitinase-like protein C25A8.4	cht-3	Secr	1.244	0.00141	
Q18943	DUF1525 domain-containing protein	CELE_D1054.10	Unclassified	1.235	0.00127	
O44400	Protein F37C4.5	F37C4.5	Unclassified	1.229	0.00295	
P55853	Small ubiquitin-related modifier	smo-1	CY, N	1.226	0.0000858	
Q27485	Histone H2A	his-35;his-3	N	1.214	0.0149	
Q9XTT3	SCAVenger receptor (CD36 family) related	scav-3	CY	1.213	0.0497	
P18948	Vitellogenin-6	vit-6	Secr	1.21	0.000535	
Q17963	WD repeat-containing protein wdr-5.1	wdr-5.1	N	1.202	0.0336	
Q20502	Histidine ammonia-lyase	haly-1	CY	1.198	0.00243	
P52717	Serine carboxypeptidase ctsa-1.1	F41C3.5	Unclassified	1.185	0.0000976	
Q23445	GTP-binding protein SAR1	sar-1	ER, CY	1.181	0.0202	
O01804	Aspartate aminotransferase	got-2.1	M	1.181	0.0155	
Q9XX57	DAF-16/FOXO Controlled, germline Tumor affecting	dct-16	Unclassified	1.178	0.00613	
G5EGK8	Serine/threonine-protein phosphatase 2A catalytic subunit	let-92	CS	1.161	0.00127	
P34382	Fatty-acid and retinol-binding protein 1	far-1	Secr	1.157	0.00743	
O02215	Gamma-cystathionase cbl-1	cbl-1	CY	1.156	0.0179	
Q21966	Aspartic protease 4	asp-4	CY, Secr	1.153	0.000188	
Q9N5M2	Prefoldin subunit 2	pdf-2	CY, ER	1.153	0.0119	
Q27473	Annexin nex-3	nex-3	Unclassified	1.143	0.0405	
O44727	Calponin-homology (CH) domain-containing protein cpn-4	cpn-4	Unclassified	1.128	0.000165	
Q93459	Cytosolic Fe-S cluster assembly factor NUBP1 homolog	F10G8.6	CY	1.126	0.0124	
Q9XWS6	Conserved secreted protein	CELE_Y62H9A.5	Unclassified	1.125	0.00612	
C0HLB4	V-type proton ATPase 16 kDa proteolipid subunit 3	vha-3	Me	1.104	0.0304	

Q9TYS3	Ferritin	ftn-2	CY	1.096	0.00742	
Q965W1	Fatty acid-binding protein homolog 9	lbp-9	CY, N	1.094	0.00677	
O01615	Acidic leucine-rich nuclear phosphoprotein 32-related protein 2	T19H12.2	Unclassified	1.093	0.0109	
Q03575	Transthyretin-like protein 5	ttr-5	Secr	1.09	0.0101	
Q0G840	15-oxoprostaglandin 13-reductase	CELE_M106.3	CY	1.088	0.0113	
Q65ZJ7	Thioredoxin domain-containing protein C32D5.8	C32D5.8	Unclassified	1.077	0.000981	
Q18705	Fibrinogen C-terminal domain-containing protein	C49C8.5	Secr	1.072	0.034	
Q17474	2-HydroxyAcyl-CoA Lyase 1	B0334.3	CY	1.067	0.0265	
Q18040	Probable ornithine aminotransferase. mitochondrial	C16A3.10	M	1.059	0.000338	=
G5ECW7	Dipeptidyl peptidase 3	dpt-1	CY	1.055	0.0138	
H9G2U2	Dauer Up-Regulated	dur-1		1.05	0.0277	
O44952	Lon protease homolog. mitochondrial	C34B2.6	M	1.049	0.0283	=
P46562	Putative aldehyde dehydrogenase family 7 member A1 homolog	alh-9	Unclassified	1.049	0.000242	
G5EEA8	Annexin nex-1	nex-1	Me	1.032	0.000025	
Q17543	Endonuclease/exonuclease/phosphatase family protein	C01B10.3	Unclassified	1.028	0.000701	
Q9U1W8	U6 snRNA-associated Sm-like protein LSm3	lsm-3	N	1.024	0.00141	
G5EET8	PUD1_2 domain-containing protein pud-1.2	pud-1.2	Unclassified	1.014	0.00849	
O01869	S10_plectin domain-containing protein	rps-10	R	1.008	0.0014	
O16202	Lysozyme-like protein 7	lys-7	Unclassified	1.006	0.0145	
Q8MQB5	TPR_REGION domain-containing protein	rmd-2	CY, M	0.995	0.0268	
Q20049	Isocitrate dehydrogenase [NAD] subunit, mitochondrial idhg-1	idhg-1	M	0.988	0.00829	
Q22562	Secreted protein	CELE_T19B10.2	Unclassified	0.987	0.0058	
Q9U315	DUF4440 domain-containing protein	CELE_Y105C5B.5	Unclassified	0.982	0.0162	
O62053	UPF0375 protein ule-4	C08F11.11	Secr	0.98	0.0304	
Q9XWV2	Putative phospholipase B-like 1	Y37D8A.2	CY	0.978	0.000299	
Q21902	DNA replication licensing factor mcm-5	mcm-5	N, CY	0.967	0.0107	
P10771	Histone 24	his-24	N, CY	0.966	0.017	
P34183	RNA ligase	hars-1	CY, M	0.965	0.00132	=
G5EES3	Protein argonaute	alg-1	CY	0.96	0.00315	
Q18212	Spliceosome RNA helicase DDX39B homolog	hel-1	N	0.952	0.00272	
Q23680	Probable V-type proton ATPase subunit F	vha-9	Me	0.95	0.000228	
G5EBJ7	Fructose-bisphosphatase	fbp-1	CY	0.945	0.000463	
O02345	Nucleotide Sugar TransPorter family	nstp-5	Me	0.945	0.0271	
G5EF37	Paralysed Arrest at Two-fold	pat-10	CY	0.943	0.00214	

Q95YA9	Adenylyl cyclase-associated protein	cas-1	CY	0.933	0.00173	
O17406	AT hook Transcription Factor family	attf-2	Unclassified	0.932	0.00148	
Q17698	DUF148 domain-containing protein nlp-77	nlp-77	Unclassified	0.928	0.000902	
A6PVA1	NudC domain-containing protein 1	C44E4.5	CY, N	0.926	0.0482	
Q27488	Proteasome subunit alpha type-2	pas-2	CY, N	0.922	0.00128	
G5ECU1	Skp1-related protein	skr-1	CY, N	0.917	0.0422	
P34556	Cyclin-dependent kinase 1	cdk-1	N, CY	0.893	0.00447	
Q21735	Probable nuclear transport factor 2	ran-4	CY	0.892	0.000832	
Q9GYF1	Troponin I 2	unc-27	CY	0.889	0.0082	
O61792	MPN domain-containing protein	rpn-8	Unclassified	0.886	0.0218	
O76371	26S protease regulatory subunit 6A	rpt-5	CY, N	0.884	0.00153	
P05690	Vitellogenin-2	vit-2	Secr	0.874	0.00247	≠
H2KYJ2	TransThyretin-Related family domain ttr-59	ttr-59	Secr	0.873	0.00587	
Q9BL60	Related to yeast Vacuolar Protein Sorting factor vps-20	vps-20	CY	0.863	0.0477	
Q20239	DUF5110 domain-containing protein	aagr-3	CY	0.862	0.00262	
Q21750	P-type domain-containing protein	aagr-2	CY, Me	0.862	0.0411	
G5ECC3	Receptor Mediated Endocytosis rme-1	rme-1	Me	0.853	0.00346	
Q22288	Transthyretin-like protein 15	ttr-15	Secr	0.853	0.00154	
Q11067	Probable protein disulfide-isomerase A6	tag-320	ER	0.851	0.00175	
P91997	Aldo_ket_red domain-containing protein CELE_F53F1.2	CELE_F53F1.2	CY	0.845	0.0229	
Q9XXI9	DNA helicase	mcm-2	N	0.842	0.00042	
Q19257	PI31_Prot_N domain-containing protein	CELE_F09E5.7	Unclassified	0.84	0.00277	
G5EC87	Glucosidase 2 subunit beta	CELE_ZK1307.8	ER	0.84	0.0197	
O76840	Papilin	mig-6	Secr	0.838	0.00195	
P62784	Histone H4	his-1	N	0.838	0.0134	
O17071	Probable 26S proteasome regulatory subunit 10B	rpt-4	CY, N	0.832	0.0163	
Q09580	Probable GMP synthase [glutamine-hydrolyzing]	gmps-1	CY	0.824	0.00939	
Q17556	NAD(P)-bd_dom domain-containing protein rml-4	rml-4	Unclassified	0.824	0.00986	
G5EG33	EGF-like domain-containing protein him-4	him-4	Secr	0.822	0.0301	
Q19655	Rieske domain-containing protein	CELE_F20D6.11	CY, ER. M	0.82	0.00448	
Q9XUS5	UTP--glucose-1-phosphate uridylyltransferase	K08E3.5	CY. ER	0.812	0.00333	
G5EFK4	ADP-Ribosylation Factor homolog	arf-3	CY	0.808	0.00221	
P30642	Eukaryotic translation initiation factor 3 subunit D	eif-3.D	CY	0.805	0.00422	
Q21763	Thioredoxin domain-containing protein CELE_R05H5.3	CELE_R05H5.3	Unclassified	0.805	0.0114	

Q18938	Probable maleylacetoacetate isomerase	gst-42	CY	0.801	0.000812	
Q93535	Protein Skeletor	CELE_F20D1.3	Unclassified	0.8	0.00332	
Q21355	Glutathione S-transferase 4	gst-4	CY	0.798	0.00468	
Q20107	Peptidyl-prolyl cis-trans isomerase fkb-1	fkb-1	CY	0.794	0.00038	
Q23280	Co-chaperone protein daf-41	ZC395.10	CY, N	0.789	0.00474	
Q22352	Aldo_ket_red domain-containing protein exc-15	exc-15	CY	0.784	0.022	
P91917	Obg-like ATPase 1	ola-1	CY	0.779	0.00798	
Q19437	CN hydrolase domain-containing protein upb-1	upb-1	CY	0.778	0.00176	
Q11176	Actin-interacting protein 1	unc-78	CS	0.777	0.000203	
Q19853	EGF-like domain-containing protein	CELE_F28B4.3	Unclassified	0.771	0.00344	
P90732	C-type LECtin	clec-41	Unclassified	0.768	0.00307	=
Q8MXS8	3-oxoacyl-[acyl-carrier-protein] reductase FabG	CELE_Y47G6A.22	Unclassified	0.768	0.012	
Q95YB2	Acyl CoA DeHydrogenase	acdh-9	Unclassified	0.767	0.03	
P48053	Uncharacterized protein C05D11.1	C05D11.1	Unclassified	0.763	0.0119	
Q19626	Probable V-type proton ATPase subunit B	vha-12	CY, Me	0.762	0.0279	
O01805	Acyl-CoA-binding protein homolog 1	acbp-1	Unclassified	0.76	0.0067	
P18334	Casein kinase II subunit alpha	kin-3	Me	0.758	0.0101	
O16266	Uncharacterized protein CELE_F40A3.6	CELE_F40A3.6	Unclassified	0.751	0.000519	
Q19007	Nucleosome Assembly Protein	nap-1	CY, N	0.75	0.0267	
Q19264	Putative deoxyribose-phosphate aldolase	F09E5.3	CY	0.748	0.00259	
Q9N5U1	Alpha-1,4 glucan phosphorylase	pygl-1	CY	0.745	0.0000123	
Q23378	TransThyretin-Related family domain ttr-48	ttr-48	Secr	0.743	0.00373	
Q9NAI5	Aldo_ket_red domain-containing protein	CELE_Y39G8B.1	CY	0.741	0.00103	
Q22515	Hypoxia up-regulated protein 1	CELE_T14G8.3	ER	0.739	0.0416	
Q22799	Dynein light chain 1, cytoplasmic	dlc-1	CS	0.738	0.00136	
V6CJ04	Protein bicaudal D homolog	bicd-1	Me	0.736	0.0196	
P34255	Probable 3-ketoacyl-CoA thiolase	B0303.3	M	0.733	0.0318	
Q9TZL8	ATP-dependent 6-phosphofructokinase 1	pfk-1	CY	0.732	0.00393	
Q9TXU7	Eukaryotic translation initiation factor 4C	eif-1.a	CY	0.732	0.00198	
O16305	Calmodulin	cmd-1	CS	0.732	0.0118	
Q2V4S2	Rab GDP dissociation inhibitor	gdi-1	CY	0.723	0.0121	
G5EF32	Nematode Polyprotein Allergen related	npa-1	Unclassified	0.722	0.0473	
Q9N588	Thymidylate synthase	tyms-1	CY,M	0.72	0.025	
D5MCR3	SUPpressor	sup-26	CY, N	0.719	0.0248	
Q9BL27	Thioredoxin domain-containing protein 17	txdc-17	CY	0.717	0.00509	
Q10039	Glycine--tRNA ligase	grs-1	CY, Secr	0.717	0.00465	

P43510	Cathepsin B-like cysteine proteinase 6	cpr-6	CY, SEcr	0.717	0.00629	
P34286	Proteasome subunit beta type-1	pbs-6	CY, N	0.715	0.00186	
Q304D5	Thioredoxin domain-containing protein erp-44.1	erp-44.1	ER	0.713	0.00339	
Q9NF11	Hypoxanthine phosphoribosyltransferase	hprt-1	CY	0.713	0.00191	
P04255	Histone H2B 1	his-11;his-4;his-48;his-41	N	0.713	0.00324	
Q18885	Transcription factor BTF3 homolog	icd-1	N, CY, M	0.712	0.0196	
P55216	Putative cystathionine gamma-lyase 2	cth-2	CY	0.707	0.00488	=
Q22494	Probable V-type proton ATPase subunit H 2	vha-15	CY, Me	0.705	0.00228	
P91020	Aldo_ket_red domain-containing protein C07D8.6	C07D8.6	CY	0.703	0.0013	=
P52275	Tubulin beta-2 chain	tbb-2	CS	0.699	0.00232	
P53014	Myosin, essential light chain	mlc-3	CY	0.696	0.00062	=
Q20660	SHSP domain-containing protein	hsp-17	Unclassified	0.695	0.00931	=
P54812	Transitional endoplasmic reticulum ATPase homolog 2	cdc-48.2	CY	0.692	0.00357	
P34690	Tubulin alpha-2 chain	tba-2	CS	0.692	0.009	
Q17348	Small nuclear ribonucleoprotein Sm D3	snr-1	N, CY	0.69	0.00747	
P52709	Threonine--tRNA ligase, cytoplasmic	tars-1	CY	0.687	0.00966	
O45060	Thioredoxin domain-containing protein	C35B1.5	Unclassified	0.686	0.0347	
Q20363	Stress-induced protein 1	sip-1	CY	0.676	0.0327	
O17643	Isocitrate dehydrogenase [NADP]	idh-2	M	0.672	0.0248	
O45552	ACetyl-CoA Acyltransferase 2 homolog	acaa-2	M	0.671	0.00467	
O18650	40S ribosomal protein S19	rps-19	R	0.667	0.0229	
Q9N4J2	Vitellogenin-3	vit-3	Secr	0.663	0.000485	
Q21193	Profilin-3	pfn-3	CS	0.659	0.0087	
Q19584	Ubiquitin fusion degradation protein 1 homolog	ufd-1	CY, N	0.656	0.0365	
P42168	Casein kinase I isoform alpha	kin-19	CY, N	0.652	0.00196	
P34517	Probable glycerol-3-phosphate dehydrogenase 2	gpdh-2	N, CS	0.649	0.00303	
Q23670	Probable DNA topoisomerase 2	K12D12.1	M	0.648	0.00275	
P52713	Probable methylmalonate-semialdehyde dehydrogenase	alh-8	M	0.645	0.0366	
Q18678	Probable serine--tRNA ligase, cytoplasmic	srs-2	CY	0.64	0.0165	
Q9N4M4	Nuclear anchorage protein 1	anc-1	N, CS	0.638	0.0221	
Q93353	Probable isocitrate dehydrogenase [NAD] subunit beta, mitochondrial	idhb-1	M	0.637	0.00867	
Q22620	Prolyl-tRNA synthetase	pars-1	CY	0.636	0.00987	
O76387	Probable peptidyl-tRNA hydrolase 2	C24G6.8	CY, M	0.632	0.00061	
G5EDV3	CSP domain-containing protein	cey-4	N	0.63	0.00791	

P42170	Ribonucleoside-diphosphate reductase small chain	rnr-2	CY	0.63	0.00646	
O17680	Probable S-adenosylmethionine synthase 1	sams-1	CY	0.625	0.00777	
O45734	Cathepsin -like	cpl-1	Secr, CY	0.625	0.0391	
Q9XW16	Profilin-1	pfn-1	CS	0.623	0.0015	
Q17967	Protein disulfide-isomerase 1	pdi-1	ER	0.621	0.00775	
Q22993	Phosphoethanolamine N-methyltransferase 2	pmt-2	Unclassified	0.617	0.0121	
Q18240	Suppressor of presenilin-2	spr-2	N	0.611	0.0252	
Q17688	Thioredoxin domain-containing protein C06A6.5	C06A6.5	ER	0.608	0.0378	
P06125	Vitellogenin-5	vit-5	Secr	0.608	0.0112	≠
A0A7I9BCB0	Amidinotransferase	ZK1307.1	Unclassified	0.605	0.00553	
Q22100	Acetyl-CoA acetyltransferase homolog, mitochondrial	kat-1	M	0.604	0.0318	
Q17361	Ubiquitin carboxyl-terminal hydrolase 14	usp-14	Unclassified	0.595	0.00253	
P30627	Globin-like protein	glb-1	CY	0.594	0.019	=
P91427	Probable phosphoglycerate kinase	pgk-1	CY	0.593	0.0275	
Q17572	Lipoprotein	C01G6.3	Unclassified	0.593	0.0199	
Q09236	Probable coatomer subunit delta	C13B9.3	CY	0.592	0.0192	
Q9TYX1	Peptidase_S9 domain-containing protein	dpf-5	CY	0.59	0.00794	
Q9BL61	RNA helicase CELE_Y65B4A.6	CELE_Y65B4A.6	CY	0.587	0.0134	
Q8MXT1	Thioredoxin domain-containing protein prdx-6	prdx-6	CY, M	0.586	0.000107	
Q27527	Enolase	enol-1	CY	0.582	0.0202	
P91871	3-oxoacyl-[acyl-carrier-protein] reductase	fasn-1	Unclassified	0.581	0.000374	
Q27371	Troponin T mup-2	mup-2	CS	0.573	0.0233	
P46563	Fructose-bisphosphate aldolase 2	aldo-2	CY	0.572	0.0277	
Q9TZS5	T-complex protein 1 subunit eta	cct-7	CY	0.571	0.00959	
B7WNA0	Pyruvate kinase pyk-1	pyk-1	CY	0.569	0.0176	
Q9XW92	V-type proton ATPase catalytic subunit A	vha-13	Me	0.568	0.0011	
Q95XR3	WD_REPEATS_REGION domain-containing protein CELE_Y39G10AR.9	CELE_Y39G10AR.9	Unclassified	0.565	0.00976	
Q05036	Heat shock protein 110	C30C11.4	CY, N	0.565	0.00167	
Q09603	ACB domain-containing protein	ech-4	M	0.564	0.00294	
Q94055	Serine--pyruvate aminotransferase	agxt-1	M	0.564	0.0104	
Q18164	Dihydropyrimidine dehydrogenase [NADP(+)]	dpyd-1	CY	0.563	0.00194	
P41996	Chondroitin proteoglycan-2	cpg-2	Me	0.561	0.0186	
Q20655	14-3-3-like protein 2	ftt-2	CY, N	0.561	0.00172	
Q20117	Glutamate--cysteine ligase	gcs-1	Unclassified	0.556	0.00118	
Q4W5P0	Homologous to Drosophila SQD (Squid) protein	sqd-1	N	0.552	0.0056	
G5EES9	ThioredoXin-Like	txl-1	CY	0.552	0.0223	
Q23258	Secreted protein	CELE_ZC373.2	Unclassified	0.551	0.00107	

O61742	26S proteasome non-ATPase regulatory subunit 4	rpn-10	CY, N	0.551	0.00217
Q21313	Laminin-like protein epi-1	epi-1	Secr	0.551	0.000363
Q19775	Protein phosphatase ppm-1.A	ppm-1	Secr	0.546	0.0124
Q19278	CoA hydrolase, mitochondrial	hach-1	M	0.545	0.0116
O62213	CSD_1 domain-containing protein	cey-1	ER, N	0.545	0.0138
Q03604	Ribonucleoside-diphosphate reductase large subunit	rnr-1	CY	0.544	0.00346
P46550	T-complex protein 1 subunit zet	cct-6	CY	0.543	0.00108
Q10454	Probable arginine kinase F46H5.3	F46H5.3	Unclassified	0.541	0.00564
H2KZK7	Dipeptidyl Peptidase Four (IV) family	dpf-3	Unclassified	0.539	0.0107
H1UBK1	Methionine aminopeptidase 2	map-2	CY	0.533	0.0104
H9G2T4	Isocitrate dehydrogenase [NADP] idh-1	idh-1	CY, M	0.533	0.002
Q19064	Periplasmic protein	CELE_E04F6.9	Unclassified	0.529	0.0221
Q9XUV0	Proteasome subunit beta type	pbs-5	CY, N	0.523	0.0125
G5EDD4	Tubulin alpha chain tba-4	tba-4	CY	0.517	0.0101
Q86NJ8	Piwi-like protein ppw-1	ppw-1	Unclassified	0.514	0.0101
Q9N5B3	Peptidase_M24 domain-containing protein	CELE_W08E12.7	Unclassified	0.512	0.00318
G5EG13	DeHydrogenases, Short chain dhs-12	dhs-12	Unclassified	0.51	0.000174
Q93615	Probable electron transfer flavoprotein subunit alpha, mitochondrial	F27D4.1	M	0.509	0.00424
Q9TXI4	Electron transfer flavoprotein subunit beta	CELE_F23C8.5	M	0.509	0.0192
P47208	T-complex protein 1 subunit delta	cct-4	CY	0.508	0.000582
O44750	Xaa-Pro aminopeptidase app-1	app-1	Unclassified	0.506	0.00119
O16249	Insulin-degrading enzyme	CELE_F44E7.4	M	0.498	0.00001
Q02332	Probable [pyruvate dehydrogenase (acetyl-transferring)] kinase, mitochondrial	pdhk-2	M	0.497	0.0218
Q19660	Precorrin-2 dehydrogenase	CELE_F21C10.10	M	0.49	0.0093
Q93576	Nucleoside diphosphate kinase	ndk-1	Me	0.482	0.00477
O16303	DNaJ domain (Prokaryotic heat shock protein) dnj-19	dnj-19	Me	0.479	0.0244
O18000	M20_dimer domain-containing protein	pes-9	CY, N	0.479	0.0168
O17607	RuvB-like 1	ruvb-1	CY, N	0.478	0.0141
P49197	40S ribosomal protein S21	rps-21	CY, ER	0.476	0.00183
U4PBY0	HECT-type E3 ubiquitin transferase	eel-1	CY, N	0.474	0.00417
P91477	Proteasome subunit beta type-2	pbs-4	CY, N	0.474	0.0055
Q93573	Translationally-controlled tumor protein homolog	tct-1	CY	0.469	0.0147
O45622	Tr-type G domain-containing protein	erfa-3	CY	0.468	0.00000124
O44995	AMP_N domain-containing protein	CELE_K12C11.1	Unclassified	0.466	0.0163

Q23382	Coiled-coil domain-containing protein 47	ccdc-47	Me	0.456	0.00115	
Q9U2Z1	Protein transport protein SEC23	sec-23	ER	0.456	0.0169	
Q18026	Kynureninase	flu-2	CY	0.451	0.00513	
G5ECS9	Calponin-homology (CH) domain-containing protein	cpt-2	M	0.445	0.00136	
Q19020	Dynein heavy chain, cytoplasmic	dhc-1	CS	0.444	0.0082	
Q22781	Acyl CoA DeHydrogenase acdh-7	acdh-7	CY, M	0.443	0.00389	
Q27481	UBA_e1_C domain-containing protein	uba-1	CY, N	0.441	0.00451	
O17954	Nematode Specific Peptide family	CELE_LLC1.2	CS	0.437	0.000524	
Q4TT88	Puromycin-sensitive aminopeptidase	pam-1	CS	0.433	0.00977	
Q8WTL6	N-alpha-acetyltransferase 16, NatA auxiliary subunit	hpo-29	CY	0.427	0.00996	
Q27245	Putative aminopeptidase W07G4.4	lap-2	CY	0.426	0.0162	
Q23449	26S proteasome non-ATPase regulatory subunit 8	rpn-12	N	0.425	0.0041	
Q9NA98	Actin-Related Proteins	arp-1	CS	0.424	0.0168	
Q9UAQ6	RAB family rab-1	rab-1	Unclassified	0.402	0.000451	
Q22235	Endoplasmin homolog	enpl-1	ER	0.397	0.00295	=
Q9TYY0	Geranylgeranyl transferase type-2 subunit alpha	CELE_M57.2	CY	0.397	0.00473	
P41988	T-complex protein 1 subunit alpha	cct-1	CY	0.393	0.00000132	
O16368	Probable 26S proteasome regulatory subunit 4	rpt-2	CY, N	0.383	0.011	
WT vs ctrl (under-represented proteins, n = 100)						
**Accession**	**Protein**	**Gene**	**Localization**	**log2FC**	**p-value**	**Notes**
P91910	Tubulin alpha-3 chain	mec-12	CY	-5.144	0.00364	
Q93425	Putative 28S ribosomal protein S5, mitochondrial	mrps-5	M	-4.194	0.000622	
O45525	Complex III subunit 8	CELE_F45H10.2	M	-3.669	0.000229	
Q17339	Female germline-specific tumor suppressor gld-1	gld-1	CY, N	-3.619	0.00000939	=
A0A486WUL5	AD domain-containing protein	CELE_M142.5	Unclassified	-3.533	0.0189	
Q18983	Proteasome adaptor and Scaffold	ecps-1	CY, N	-3.331	0.00264	
O16298	ATP synthase subunit epsilon, mitochondrial	hpo-18	M	-3.26	0.000157	=
Q23552	Phosphoethanolamine N-methyltransferase 1	pmt-1	CY	-3.25	0.000194	
O62327	Glutathione peroxidase 2	gpx-2	CY	-3.076	0.0288	
Q9BL34	Cytochrome OXidase assembly protein	cox-6b	M	-2.666	0.0458	
P91913	60S acidic ribosomal protein P1	rla-1	R	-2.484	0.0000105	
Q966I8	Proteasome subunit beta type pbs-1	pbs-1	CY, N	-2.48	0.018	
Q21633	Ubiquitin-conjugating enzyme E2 ubc-18	ubc-18	N	-2.426	0.0000291	
Q7YZW5	Protein vem-1	vem-1	Me	-2.381	0.00191	
Q9N4G8	Receptor expression-enhancing protein	yop-1	Me	-2.376	0.0498	
Q22285	Transthyretin-like protein 46	ttr-46	Secr	-2.372	0.0161	=

C6KRN1	Suppressor of aph-1	sao-1	CY	-2.304	0.00014	=
P91276	Importin subunit alpha-2	ima-2	CY, N	-2.279	0.000218	
Q22966	C-type lectin domain-containing protein	clec-1	Me	-2.119	0.0232	
Q9XWU9	Secreted protein	CELE_Y37D8A.19	Unclassified	-2.098	0.0015	
Q95X21	Cytosolic purine 5'-nucleotidase	CELE_Y71H10B.1	Unclassified	-2.084	0.000218	
Q22972	Peptidase A1 domain-containing protein asp-13	asp-13	CY	-2.053	0.00598	
Q9N538	DeHydrogenases, Short chain dhs-9	dhs-9	Unclassified	-1.982	0.0102	
G5EBI0	Endoplasmic reticulum transmembrane protein	4D656	ER	-1.97	0.000078	
G5EDC6	ARF-Like	arl-8	CY	-1.924	0.000105	
Q19286	Intermediate filament protein ifb-2	ifb-2	CY	-1.918	0.0012	=
O44954	Succinate dehydrogenase [ubiquinone] flavoprotein subunit, mitochondrial	sdha-2	M	-1.883	0.00887	
O76449	Protein F37C4.5	CELE_ZK1055.7	Unclassified	-1.859	0.00504	
Q23307	Uncharacterized protein ZC412.3	ZC412.3	Secr	-1.83	0.0182	
Q9GZE9	Lipid droplet localized protein	ldp-1	Me, CY	-1.813	0.00511	
O45499	40S ribosomal protein S26	rps-26	CY, ER	-1.796	0.0327	
Q65XX1	RNA helicase vbh-1	vbh-1	CY, N	-1.793	0.00361	
G5EBH7	CALUmenin (Calcium-binding protein) homolog	calu-1	ER	-1.79	0.000484	
O61848	CaLponIn-liKe proteins clik-3	clik-3	CS	-1.766	0.0000402	
O62220	Acidic leucine-rich nuclear phosphoprotein 32-related protein 1	F33H2.3	Unclassified	-1.754	0.00559	
H2KYX4	DUF3810 domain-containing protein	C23H3.2	Me	-1.725	0.00562	
Q21544	Pyrroline-5-carboxylate reductase	pycr-1	Unclassified	-1.724	0.0133	
Q93204	Glutathione peroxidase	gpx-5	Secr	-1.722	0.0395	
P34460	Probable elongation factor 1-beta/1	eef-1B.1	CY	-1.676	0.00303	
O45391	Gamma-cystathionase	cth-1	CY	-1.652	0.0000796	=
Q18496	Acetyl-coenzyme A synthetase	acs-19		-1.641	0.00192	=
Q95QQ4	5-aminoimidazole-4-carboxamide ribonucleotide formyltransferase	atic-1	CY	-1.589	0.00693	=
H2KZA3	ZM domain-containing protein	pqn-22	Unclassified	-1.581	0.0463	=
Q10576	Prolyl 4-hydroxylase subunit alpha-1	dpy-18	ER	-1.567	0.00361	
G5ECL3	Pre-RNA processing 21	prp-21	N	-1.548	0.00166	
G5EFJ3	NADAR domain-containing protein	C08E8.4	Unclassified	-1.532	0.0327	
P19826	Vinculin	deb-1	CY, Me	-1.528	0.0463	=
Q9TYW1	Vacuolar H ATPase vha-19	vha-19	Me	-1.515	0.0216	
H2KZJ5	GLutaRedoXin	glrx-3	CY, N	-1.506	0.0125	
Q95ZS5	Leucine-rich repeat domain, L domain-like	CELE_F56A8.3	Me	-1.498	0.0398	=

Q09545	Succinate dehydrogenase [ubiquinone] iron-sulfur subunit, mitochondrial	sdhb-1	M	-1.484	0.0081	
O18693	Fatty Acid CoA Synthetase family	acs-2	M	-1.474	0.00254	=
Q17974	Leucine-rich PPR motif-containing protein, mitochondrial	mma-1	M, N	-1.462	0.00322	
Q9NEZ8	Enoyl-CoA Hydratase	ech-7	M	-1.448	0.0029	=
Q09665	Troponin C. isoform 2	tnc-2	Unclassified	-1.443	0.0341	
Q93831	NADH dehydrogenase [ubiquinone] 1 beta subcomplex subunit 10	CELE_F59C6.5	M	-1.427	0.00341	
Q9XVM0	LETM1 (Leucine zipper, EF-hand, TransMembrane mitochondrial protein) homolog letm-1	letm-1	M	-1.411	0.0391	
O18229	Putative 6-phosphogluconolactonase	Y57G11C.3	N	-1.41	0.0342	
P90789	NADH dehydrogenase [ubiquinone] 1 beta subcomplex subunit 7	D2030.4	M	-1.408	0.000066	
Q09289	Proteasomal ubiquitin receptor ADRM1 homolog	C56G2.7	CY, N	-1.387	0.05	
Q21551	MICOS complex subunit MIC19	chch-3	M	-1.378	0.017	=
H2L049	TDP43_N domain-containing protein	CELE_F46H5.7	CY, N	-1.336	0.00501	
O45551	Eukaryotic translation initiation factor 4E-1	ife-1	CY	-1.335	0.0181	
Q93315	Cytochrome b5 heme-binding domain-containing protein	cytb-5.1	CY	-1.332	0.0236	
P34466	Clustered mitochondria protein homolog	clu-1	CY	-1.322	0.0388	
A0A6V7QYI0	AQuaPorin or aquaglyceroporin related	aqp-7	Me	-1.301	0.00361	=
O45218	Alkyldihydroxyacetonephosphate synthase	ads-1	CY	-1.299	0.00783	
Q21746	Small Glutamine-rich Tetratrico repeat protein	sgt-1	Me	-1.271	0.00286	
Q19519	Vacuolar protein-sorting-associated protein 36	vps-36	CY	-1.264	0.00673	
Q965G5	MAP kinase-activated protein kinase mak-2	mak-2	CY, N	-1.261	0.0319	
Q06561	Basement membrane proteoglycan	unc-52	Me	-1.242	0.00112	
P98080	Cytochrome b-c1 complex subunit 1, mitochondrial	ucr-1	M	-1.24	0.000694	
Q86NE0	Peptidase A1 domain-containing protein asp-2	asp-2	CY	-1.234	0.000152	
Q9XWG2	Complex I-B8	CELE_Y63D3A.7	M	-1.201	0.00402	
O17268	Late endosomal/lysosomal adaptor and MAPK and MTOR activator 5	lmtr-5	CY	-1.2	0.0226	
D6RYD3	Copper transporter	CELE_Y58A7A.1	Me	-1.127	0.0185	
P34369	RNA-splicing factor 8 homolog	prp-8	N	-1.127	0.0126	
Q9TZ90	Mitochondrial Ribosomal Protein, Large	mrpl-40	M	-1.111	0.045	
Q93618	Zinc finger CCCH domain-containing protein 15 homolog	F27D4.4	CY	-1.09	0.00253	

G5EDD1	Ubiquinol-Cytochrome c oxidoReductase complex	ucr-2.1	Unclassified	-1.081	0.0381	
Q20950	Pept_C1 domain-containing protein	cpr-9	CY, Secr	-1.06	0.00844	
P37806	Protein unc-87	unc-87	CS	-1.056	0.000594	
Q21443	Lamin-1	lmn-1	N	-1.055	0.00725	
O45599	Chitin-binding domain protein cbd-1	cbd-1	Secr	-1.033	0.000134	=
Q9TYL2	Delta(3,5)-Delta(2,4)-dienoyl-CoA isomerase, mitochondrial	CELE_Y25C1A.13	M	-0.986	0.0393	
G5EE48	TransThyretin-Related family domain ttr-50	ttr-50	Secr	-0.986	0.0464	
B3WFV9	Anion exchange protein	abts-1	Me	-0.978	0.0466	
Q9U307	Glutamine synthetase	gln-3	CY	-0.971	0.0256	
Q17872	Nuclear Pore complex Protein npp-23	npp-23	N	-0.965	0.0166	
G5EFH8	PALP domain-containing protein	cbs-1	CY	-0.954	0.00041	
Q19749	Dihydrolipoyllysine-residue acetyltransferase component of pyruvate dehydrogenase complex, mitochondrial	dlat-1	M	-0.922	0.00579	
Q21930	60S ribosomal protein L28	rpl-28	R	-0.916	0.00467	
Q22508	Uncharacterized protein tag-18	tag-18	Unclassified	-0.911	0.0325	
Q9N5Y2	Probable very-long-chain enoyl-CoA reductase art-1	art-1	ER	-0.905	0.0315	
P91398	CSD_1 domain-containing protein cey-3	cey-3	N	-0.891	0.000837	
Q9NEW6	Probable splicing factor, arginine/serine-rich 3	rsp-3	N	-0.891	0.0115	
Q9BL83	Vesicle-fusing ATPase	vps-4	CY	-0.881	0.0384	
Q19969	Importin subunit alpha-3	ima-3	CY, N	-0.868	0.0056	
Q9XW01	Tetratricopeptide repeat protein 38	CELE_Y54G11A.7	Unclassified	-0.864	0.00622	
G5EEM9	Intracellular phospholipase	ipla-1	CY, ER, N	-0.86	0.0221	
Q22053	rRNA 2-O-methyltransferase fibrillarin	fib-1	N	-0.858	0.0404	
P34455	Probable aconitate hydratase, mitochondrial	aco-2	M	-0.848	0.00358	=
G5EFS5	B30.2/SPRY domain-containing protein	CELE_F45D11.15	Unclassified	-0.839	0.0317	
O44512	Cytochrome b-c1 complex subunit Rieske, mitochondrial	isp-1	M	-0.834	0.0119	
Q19200	Stomatin-1	sto-1	Me	-0.833	0.0016	
O44441	ATPase inhibitor mai-2, mitochondrial	mai-2	M	-0.824	0.00354	
Q20222	Fatty acid-binding protein homolog 3	lbp-3	Secr	-0.821	0.0191	
Q9N4Y8	NADH-ubiquinone oxidoreductase 75 kDa subunit, mitochondrial	nuo-5	M	-0.819	0.0186	
O62246	Coatomer subunit epsilon	cope-1	CY, M	-0.794	0.0435	
Q9TYV5	26S rRNA (cytosine-C(5))-methyltransferase nsun-1	nol-1	N	-0.794	0.0421	
V6CLI5	AMP-binding domain-containing protein	acs-13	ER	-0.787	0.0224	

O02267	Complex I-B14.5a	CELE_F45H10.3	M	-0.778	0.000984	=
Q9BKU4	Mitochondrial prohibitin complex protein 1	phb-1	M	-0.776	0.0213	
P91306	CSD_1 domain-containing protein cey-2	cey-2	N	-0.774	0.00222	
P34519	Putative tricarboxylate transport protein, mitochondrial	K11H3.3	M	-0.75	0.00439	
P48162	60S ribosomal protein L23a 1	rpl-25.1	R	-0.744	0.0017	
G5EDY2	Tryptophanyl-tRNA synthetase	wars-1	CY	-0.737	0.00911	
Q18224	CCR4-NOT transcription complex subunit 9	ntl-9	CY	-0.727	0.00363	
P52554	Probable prefoldin subunit 6	pfd-6	CY	-0.724	0.0238	
Q17334	Alcohol dehydrogenase 1	sodh-1	CY	-0.714	0.00495	=
Q94230	Transcriptional activator plp-1	plp-1	N	-0.701	0.0347	
G5EC98	CTP synthase	ctps-1	CY	-0.699	0.0241	
H9G340	MercaptoPyruvate SulfurTransferase homolog	mpst-3	M	-0.699	0.00426	
Q7Z1Q3	Aldedh domain-containing protein	alh-12	Unclassified	-0.685	0.0237	
O17352	Gal_mutarotas_2 domain-containing protein	aagr-4	Unclassified	-0.647	0.0238	
O45679	Bifunctional L-3-cyanoalanine synthase/cysteine synthase	cysl-2	CY	-0.641	0.017	
Q09629	Probable adenylate kinase isoenzyme ZK673.2	ZK673.2	CY	-0.634	0.025	
Q7JNG1	ATP synthase subunit O, mitochondrial	atp-3	M	-0.621	0.00667	
Q93235	Sodium/potassium-transporting ATPase subunit beta-1	nkb-1	Me	-0.608	0.0381	
Q9U2F2	Carnitine O-palmitoyltransferase	cpt-1	M	-0.605	0.011	
Q9N3T5	AFG3-like protein spg-7	spg-7	M	-0.603	0.0109	
P90868	Proteasome subunit beta	pbs-7	CY, N	-0.59	0.00826	
P91390	Dolichyl-diphosphooligosaccharide--protein glycosyltransferase subunit	ostd-1	ER	-0.583	0.0000616	
P54688	Branched-chain-amino-acid aminotransferase, cytosolic	bcat-1	CY	-0.554	0.00236	
O45011	Arginine kinase CELE_W10C8.5	CELE_W10C8.5	Unclassified	-0.55	0.00385	
G5EEK9	V-type proton ATPase subunit a	vha-5	Me	-0.545	0.00805	
P40614	Phosphate carrier protein, mitochondrial	F01G4.6	M	-0.503	0.00309	
Q18066	Disorganized muscle protein 1	dim-1	CY	-0.482	0.0135	
P0DM42	Actin-3	act-3;act-1	CS	-0.408	0.00376	
P12844	Myosin-3	myo-3	CY	-0.393	0.0103	

Table S2. MS- identified and quantified metabolites. Results of the unpaired non-parametric Mann-Withney test applied to each class of quantified metabolites. = Same direction of change; ≠ different direction of change; + Significantly altered in one comparison; ++ Significantly altered in both comparison

	Metabolite	Discovery?		q value		Mean rank diff.		Notes
		WT vs Ctrl	D76N vs Ctrl	WT vs Ctrl	D76N vs Ctrl	WT vs Ctrl	D76N vs Ctrl	
Aminoacids	Ala	Yes	Yes	0.000366	0.000075	-8.556	-9.000	=, ++
	Arg	Yes	No	0.000823	0.545701	8.111	-1.667	≠, +
	Asn	No	Yes	0.214968	0.000075	-3.444	9.000	≠, +
	Asp	No	Yes	0.214968	0.000075	3.444	-9.000	≠, +
	Cys	No	Yes	0.474455	0.000075	1.889	9.000	= +
	Gln	Yes	Yes	0.000118	0.000759	-9.000	-8.111	=, ++
	Glu	Yes	Yes	0.000118	0.000075	-9.000	-9.000	=, ++
	Gly	Yes	Yes	0.000118	0.006053	9.000	6.889	=, ++
	His	Yes	Yes	0.000118	0.000075	-9.000	-9.000	=, ++
	Ile	Yes	Yes	0.001303	0.000075	-7.889	9.000	≠, ++
	Leu	Yes	Yes	0.000366	0.000075	-8.556	9.000	≠, ++
	Lys	No	No	0.083368	0.247132	4.778	3.222	=
	Met	Yes	No	0.000118	0.261621	-9.000	3.000	≠, +
	Phe	Yes	No	0.000118	0.013266	-9.000	6.333	≠, ++
	Pro	No	Yes	0.044955	0.007514	5.444	6.778	=, +
	Ser	Yes	Yes	0.000118	0.000075	-9.000	-9.000	=, ++
	Thr	No	No	0.247132	0.016696	3.222	-6.111	≠
	Trp	Yes	Yes	0.000897	0.000759	-8.111	8.111	≠, ++
	Tyr	No	Yes	0.406850	0.000075	-2.333	9.000	≠, +
	Val	Yes	Yes	0.006139	0.000075	-7.000	9.000	≠, ++
Aminoacids Related	1-Methylhistidine	Yes	No	0.000098	0.237402	9.000	-3.556	≠, +
	α-Aminobutyric acid	Yes	Yes	0.000098	0.001407	-9.000	8.000	≠, ++
	α-Aminoadipic acid	Yes	Yes	0.000098	0.005471	-9.000	-7.111	=, ++
	Anserine	Yes	No	0.000924	0.237402	8.222	-3.333	≠, +
	β-Aminobutyric acid	Yes	Yes	0.000098	0.005471	-9.000	7.000	≠, ++
	Betaine	Yes	Yes	0.000098	0.000130	9.000	9.000	=, ++
	cis -4-Hydroxyproline	Yes	Yes	0.000098	0.000130	9.000	-9.000	≠, ++
	Carnosine	Yes	Yes	0.000098	0.000447	-9.000	8.556	≠, ++
	Citrulline	Yes	Yes	0.000174	0.000488	-8.889	8.556	≠, ++
	Dihydroxy-phenylalanine	No	No	0.010942	0.325042	6.333	2.667	=
	Homoarginine	Yes	Yes	0.002280	0.000608	7.667	-8.333	≠,++
	Homocysteine	No	No	0.233820	0.077917	3.222	4.889	=
	Kynurenine	Yes	No	0.000234	0.567503	-8.778	-1.444	=, +
	Methionine sulfoxide	No	No	0.372974	0.395084	2.333	2.333	=
	Ornithine	Yes	Yes	0.005415	0.000130	7.000	-9.000	≠, ++
	Sarcosine	Yes	Yes	0.002645	0.000130	-7.444	-9.000	=, ++
	Symmetric dimethylarginine	No	No	0.018074	0.208233	6.000	4.000	=

	trans -4-Hydroxyproline	Yes	Yes	0.000098	0.000130	9.000	-9.000	≠, ++
	Taurine	No	Yes	0.056227	0.000130	5.000	-9.000	≠, +
Vitamins & Cofactors	Choline	Yes	Yes	0.000041	0.000041	9.000	9.000	=, ++
Biogenic Amines	Dopamine	Yes	Yes	0.000123	0.000082	9.000	9.000	=, ++
	GABA	Yes	Yes	0.000165	0.001111	8.778	7.889	=, ++
	Putrescine	Yes	Yes	0.000123	0.000082	9.000	9.000	=, ++
	Serotonin	Yes	No	0.002715	0.082353	7.000	4.000	=, +
	Spermidine	No	Yes	0.181859	0.000082	3.444	9.000	= +
	Spermine	Yes	No	0.002715	0.059729	7.222	-5.000	≠, +
Carboxylic Acids	Aconitic acid	No	Yes	0.044118	0.000062	5.000	9.000	=, +
	Hippuric acid	No	Yes	0.816865	0.000062	0.6667	-9.000	≠, +
	Lactic acid	Yes	Yes	0.000082	0.000041	9.000	9.000	=, ++
	3-Hydroxyglutaric acid	Yes	Yes	0.000123	0.000165	9.000	8.556	=, ++
	Succinic acid	No	Yes	0.489428	0.000041	1.889	9.000	=, +
Fatty Acids	Eicosapentaenoic acid	Yes	No	0.006911	0.328301	7.667	-2.556	≠, +
	Myristic acid	No	Yes	0.931427	0.000346	0.3333	-8.333	≠, +
	Palmitic acid	No	Yes	0.680214	0.000247	2.556	-8.778	≠, +
	Stearic acid	No	Yes	0.876528	0.000247	-1.000	-8.556	=, +
	Eicosenoic acid	No	Yes	0.570753	0.000247	-3.444	-8.556	=, +
	Eicosadienoic acid	No	Yes	0.876528	0.000247	-1.333	-9.000	=, +
Amine Oxides	Trimethylamine N-oxide	No	No	0.619910	0.070588	2	-4.333	≠
Bile Acids	Glycocheno-deoxycholic acid	No	No	0.205882	0.205882	-3.000	-3.000	=
Indole derivatives	3-Indolepropionic acid	No	No	>0.99999	0.470588	-1.000	-2.000	=
Nucleobases Related	Hypoxanthine	Yes	Yes	0.005430	0.000082	6.778	-9.000	≠, ++
	Xantine	Yes	No	0.005430	0.248046	7.000	3.000	=, +

Table S3. Results of the joint pathway analysis of proteomics and metabolomics data.

WT vs ctrl				
**Pathway**	**Hits**	**Match status**	**-log10(p)**	**Impact**
Glycolysis or Gluconeogenesis	Lactate; Glucose 1-P; beta-Glucose; alpha-Glucose; alh-9; alh-12; sodh-1; F23B12.5; pyk-1; enol-1; aldo-2; pkf-1; fbp-1; pgk-1; acs-19	15/52	5.3115	0.96
Arginine and proline metabolism	Arginine; Putrescine; Glutamate; Ornithine; GABA; Hydroxyproline; F46H5.3; CELE_W10C8.5; alh-9; alh-12; got-2.1; pycr-1; dpy-18; C16A3.10	14/48	5.0443	0.70
Aminoacyl-tRNA biosynthesis	Histidine; Phenylalanine; Arginine; Glutamine; Glycine; Methionine; Valine; Alanine; Isoleucine; Leucine; Glutamate; hrs-1; grs-1; srs-2; trs-1; wars-1; pars-1	17/70	4.8428	0.36
Glutathione metabolism	Glycine; Glutamate; Ornithine; Putrescine; lap-2; gpx-5; gpx-2; idh-2; idh-1; gcs-1; gst-7; gst-4; rnr-2; rnr-1	14/50	4.8155	0.65
Histidine metabolism	Histidine; Carnosine; Anserine; haly-1; alh-9; alh-12	6/13	3.6193	0.58
Arginine biosynthesis	Glutamate; Glutamine; Ornithine; gln-3; got-2.1	5/12	2.8338	1.00
beta-Alanine metabolism	ech-7; hach-1; alh-8; alh-9; alh-12; upb-1; dpyd-1	7/24	2.7312	0.87
Valine, leucine and isoleucine degradation	Valine; Isoleucine; Leucine; kat-1; ech-7; acdh-7; bcat-1; B0303.3; acaa-2; acdh-9; alh-9; alh-12; alh-8; hach-1	14/84	2.3086	0.54
Valine, leucine and isoleucine biosynthesis	Leucine; Isoleucine; Valine; bcat-1	4/12	1.9445	1.09
Citrate cycle (TCA cycle)	idh-2; idh-1; idhb-1; idhg-1; sdha-2; sdhb-1; aco-2; F23B12.5	8/42	1.8275	0.73
Fatty acid degradation	acdh-7; kat-1; B0303.3; acaa-2; ech-7; ech-4; sodh-1; alh-9; alh-12; acs-2; acs-13; cpt-1; cpt-2	13/92	1.588	1.38
Glycine, serine and threonine metabolism	Choline; Glycine; Sarcosine; Betaine; alh-9; agxt-1; cbs-1; cth-1; cth-2	9/55	1.5855	0.87
Pyruvate metabolism	Lactate; pyk-1; acs-19; alh-9; alh-12; kat-1; F23B12.5	7/38	1.5756	0.65
Drug metabolism - other enzymes	hprt-1; gmps-1; dpyd-1; upb-1; rnr-2; rnr-1; ndk-1; gst-7; gst-4	9/56	1.5388	0.31
Alanine, aspartate and glutamate metabolism	Alanine; Glutamine; Glutamate; GABA; got-2.1; agxt-1; gln-3	7/39	1.5185	0.76
Galactose metabolism	Alpha-Glucose; Glucose-1P; pfk-1; CELE_Y39G8B.1; K08E3.5	5/25	1.3707	0.87
Nitrogen metabolism	Glutamine; Glutamate; gln-3	3/11	1.2981	0.70
Glyoxylate and dicarboxylate metabolism	Glycine; Glutamate; Glutamine; aco-2; kat-1; agxt-1; gln-3; acs-19	8/53	1.272	0.44
Tryptophan metabolism	Kynurenine; Tryptophan; Serotonin; kat-1; ech-7; alh-10; flu-2; alh-9	8/58	1.0812	0.37
Cysteine and methionine metabolism	Methionine; cth-1; C23H3.2; cbs-1; sams-1; cysl-2; got-2.1; gcs-1; bcat-1	9/69	1.0444	0.62
Butanoate metabolism	GABA; Glutamate; kat-1; ech-7	4/23	0.98559	0.54
Fructose and mannose metabolism	Alpha-glucose; CELE_Y39G8B.1; pfk-1; aldo-2; fbp-1	5/32	0.98364	0.64
D-Glutamine and D-glutamate metabolism	Glutamate; Glutamine	2/8	0.89343	0.57
Phenylalanine, tyrosine and tryptophan biosynthesis	Phenylalanine; got-2.	2/9	0.8066	1.62
Starch and sucrose metabolism	Glucose-1P; Trehalose; K08E3.5; pygl-1	4/28	0.75166	0.33
Lysine degradation	alpha-Aminoadipic acid; alh-12; alh-9; ech-7; kat-1	5/38	0.74859	0.30
Ascorbate and aldarate metabolism	alh-9; alh-12	2/10	0.73184	0.44
Tyrosine metabolism	Dopamine; sodh-1; gst-42; got-2.1; tyr-4	5/40	0.68429	0.51
Arachidonic acid metabolism	gpx-5; gpx-2; ZC395.10	3/20	0.68159	0.31
Pantothenate and CoA biosynthesis	Valine; upb-1; dpyd-1; bcat-1	4/30	0.67644	0.41

Phenylalanine metabolism	Phenylalanine; got-2.1	2/12	0.60931	0.91
Drug metabolism - cytochrome P450	gst-7; gst-4; sodh-1	3/22	0.59919	0.38
Pyrimidine metabolism	Glutamine; rnr-2; rnr-1; ndk-1; ctps-1; CELE_Y71H10B.1; dpyd-1; upb-1; tyms-1;	9/88	0.58582	0.61
Pentose phosphate pathway	F09E5.3; aldo-2; pfk-1; fbp-1; Y57G11C.3	5/45	0.54732	0.32
Propanoate metabolism	alh-8; acs-19; hach-1; kat-1; ech-7	5/45	0.54732	0.50
Glycerolipid metabolism	CELE_Y39G8B.1; alh-9; alh-12	3/25	0.49634	0.33
Pentose and glucuronate interconversions	Glucose-1P; CELE_Y39G8B.1; K08E3.5	3/26	0.46662	0.44
Purine metabolism	Glucose-1P; alpha-Glucose; Hypoxanthine; rnr-2; rnr-1; atic-1; ndk-1; ZK673.2; CELE_Y71H10B.1; gmps-1; hprt-1; pyk-1	12/142	0.34062	0.72
Selenocompound metabolism	Alanine; cth-1; cth-2	3/32	0.3244	0.16
Synthesis and degradation of ketone bodies	kat-1	1/8	0.31423	0.57
Amino sugar and nucleotide sugar metabolism	Glucose-1P; alpha-Glucose; rml-4; K08E3.5; cht-1	5/60	0.2791	0.30
Thiamine metabolism	ZK673.2	1/11	0.22267	0.20
One carbon pool by folate	tyms-1; atic-1	2/26	0.20401	0.32
Retinol metabolism	sodh-1	1/17	0.12092	0.25
Glycerophospholipid metabolism	PhosphoCholine; Choline; GlyceroPhosphoCholine; gpdh-2	4/69	0.089342	0.19
Metabolism of xenobiotics by cytochrome P450	gst-7; gst-4; sodh-1	3/56	0.077202	0.51
Ether lipid metabolism	ads-1	1/22	0.07562	0.09
Sulfur metabolism	cysl-2	1/22	0.07562	0.19
Fatty acid elongation	ech-7; B0303.3; acaa-2; art-1	4/73	0.071804	0.67
Terpenoid backbone biosynthesis	kat-1	1/30	0.036906	0.14
N-Glycan biosynthesis	aagr-3; aagr-4; ostd-1	3/68	0.03666	0.12
Porphyrin and chlorophyll metabolism	Glutamate	1/32	0.030957	0.03
Nicotinate and nicotinamide metabolism	CELE_Y71H10B.1	1/33	0.028362	0.12
Folate biosynthesis	CELE_Y39G8B.1	1/51	0.005969	0.08
Inositol phosphate metabolism	alh-8	1/53	0.0050227	0.04
Fatty acid biosynthesis	fasn-1; acs-2; acs-13	3/103	0.0035329	1.20

D76N vs ctrl

Pathway	**Hits**	**Match status**	**-log10(p)**	**Impact**
Aminoacyl-tRNA biosynthesis	Asparagine; Histidine; Glutamine; Cysteine; Glycine; Aspartate; Valine; Alanine; Isoleucine; Leucine; Tryptophan; Tyrosine; Proline; Glutamate; hrs-1; yrs-2	16/70	9.8987	0.26
Arginine and proline metabolism	Putrescine; Proline; Glutamate; Ornithine; GABA; Spermidine; Hydroxyproline; argk-1; C16A3.10	9/48	4.827	0.49
Alanine, aspartate and glutamate metabolism	Aspartate; Alanine; Glutamine; Glutamate; GABA; Asparagine; Succinate; pyr-1	8/39	4.627	0.90
Butanoate metabolism	GABA; Glutamate; Succinate; C05C10.3; ech-7	5/23	3.15	0.60
Glutathione metabolism	Glycine; Glutamate; Cysteine; Ornithine; Putrescine; Spermidine; gst-7	7/50	3.0108	0.41
Glyoxylate and dicarboxylate metabolism	Aconitic Acid; Glycine; Glutamate; Succinate; Glutamine; aco-2; acs-19	7/53	2.8558	0.38
Glycine, serine and threonine metabolism	Choline; Glycine; Sarcosine; Betaine; Cysteine; cth-1; cth-2	7/55	2.7587	0.52
Valine, leucine and isoleucine biosynthesis	Leucine; Isoleucine; Valine	3/12	2.2116	0.36
Arginine biosynthesis	Glutamate; Glutamine; Ornithine	3/12	2.2116	0.64

Propanoate metabolism	Succinate; beta-Alanine; Propanoate; acs-19; ech-7	5/45	1.8304	0.45
D-Glutamine and D-glutamate metabolism	Glutamate; Glutamine	2/8	1.5746	0.57
Taurine and hypotaurine metabolism	Cysteine; Taurine	2/10	1.3869	0.33
beta-Alanine metabolism	beta-Alanine; Aspartate; ech-7	3/24	1.3696	0.48
Nitrogen metabolism	Glutamine; Glutamate;	2/11	1.309	0.50
Histidine metabolism	Histidine; Carnosine	2/13	1.1755	0.17
Pantothenate and CoA biosynthesis	Valine; Cysteine; beta-Alanine	3/30	1.1274	0.14
Selenocompound metabolism	Alanine; cth-1; cth-2	3/32	1.0603	0.16
Glycolysis or Gluconeogenesis	Lactate; Glucose-1P; sodh-1; acs-19	4/52	1.0435	0.20
Valine, leucine and isoleucine degradation	Valine; Isoleucine; Leucine; C05C10.3; ech-7	5/84	0.84803	0.22
Tyrosine metabolism	Dopamine; Tyrosine; sodh-1	3/40	0.84013	0.41
Citrate cycle (TCA cycle)	Succinate; Aconitic acid; aco-2	3/42	0.79456	0.44
Drug metabolism - cytochrome P450	gst-7; sodh-1	2/22	0.78511	0.38
Ubiquinone and other terpenoid-quinone biosynthesis	Tyrosine	1/8	0.62488	0.14
Synthesis and degradation of ketone bodies	C05C10.3	1/8	0.62488	0.57
Phenylalanine, tyrosine and tryptophan biosynthesis	Tyrosine	1/9	0.5806	0.62
Thiamine metabolism	Cysteine	1/11	0.50709	0.10
Phenylalanine metabolism	Tyrosine	1/12	0.47606	0.09
Lysine degradation	alpha-Aminoadipic acid; ech-7	2/38	0.44111	0.08
Pyruvate metabolism	Lactate; acs-19	2/38	0.44111	0.27
Fatty acid degradation	Hexadecanoic acid; ech-7; sodh-1; acs-2	4/92	0.43736	0.42
Cysteine and methionine metabolism	Cysteine; cth-1; cth-2	3/69	0.39313	0.34
Retinol metabolism	sodh-1	1/17	0.35805	0.25
Sulfur metabolism	Cysteine	1/22	0.27841	0.05
Metabolism of xenobiotics by cytochrome P450	gst-7; sodh-1	2/56	0.24924	0.51
Pyrimidine metabolism	Glutamine; beta-Alanine; pyr-1	3/88	0.24504	0.11
Galactose metabolism	Glucose-1P	1/25	0.24185	0.25
Tryptophan metabolism	Tryptophan; ech-7	2/58	0.23446	0.10
Pentose and glucuronate interconversions	Glucose-1P	1/26	0.23106	0.04
One carbon pool by folate	atic-1	1/26	0.23106	0.16
Starch and sucrose metabolism	Glucose-1P	1/28	0.21126	0.15
Porphyrin and chlorophyll metabolism	Glutamate	1/32	0.17758	0.03
Nicotinate and nicotinamide metabolism	Aspartate	1/33	0.1702	0.03
Fatty acid biosynthesis	Hexadecanoic acid; Tetradecanoic acid; acs-2	3/103	0.16876	0.09
Fatty acid elongation	Hexadecanoic acid; ech-7	2/73	0.14923	0.39
Purine metabolism	Glutamine; Hypoxanthine; Inosine; atic-1	4/142	0.14868	0.13
Drug metabolism - other enzymes	gst-7	1/56	0.068387	0.04
Amino sugar and nucleotide sugar metabolism	Glucose-1P	1/60	0.058791	0.10
Glycerophospholipid metabolism	Choline	1/69	0.042	0.06

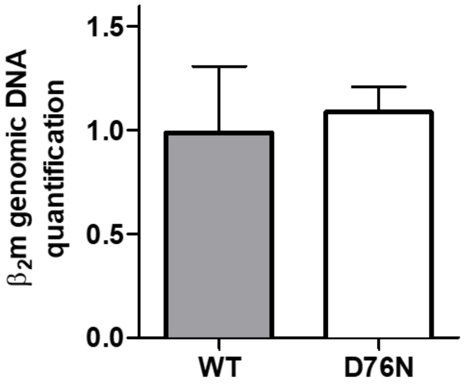

Figure S1. Relative quantification by qPCR of D76N and WT β_2-m DNA extracted from the two *C. elegans* strains. Genomic DNA was extracted from 5'000 nematode's samples of both WT β_2-m and D76N β_2-m strains after lysis in 5 volumes of worm genomic DNA lysis buffer (10mM Tris pH 7.5, 2mM EDTA, 0.5% SDS) and proteinase K 0,1 mg/ml. Organic extraction was performed with phenol/chloroform/isoamyl alcohol and qPCR on DNA samples extracted from both strains was performed using a Quantifast SYBR Green PCR (QIAGEN, 204054) together with β_2-m and *cdc42 (Cell Division Cycle*) primers. Relative quantification was performed using Cdc-42 as housekeeping gene. Data shown in the graph correspond to normalized Ct values of six replicates from two independent experiments. β_2-m gene quantification of wildtype and mutated D76N protein showed no significant differences between the two strains according to the Mann-Whitney test (GraphPad Prism).

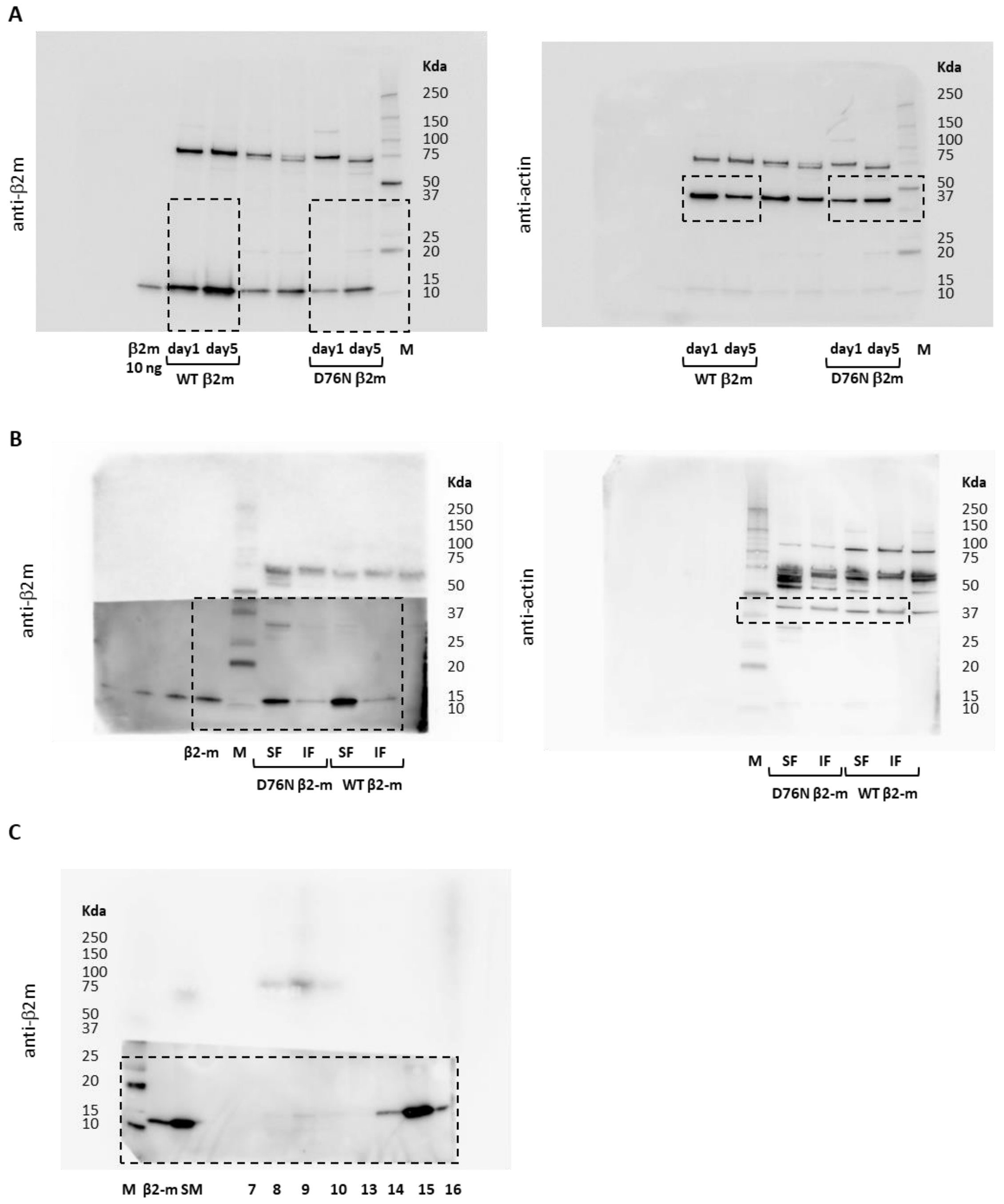

Figure S2. A) and B) Western blots for detection of β2-m and actin. Delimited portions (dotted squares) are used in Figure 1C and in Figure 2A respectively. (M= Molecular weight standard: Precision Plus Western C, BioRad). C) Western blots for detection of β2-m. Delimited portion (dotted squares) is used in Figure 2C (M= Molecular weight standard: Precision Plus Western C, BioRad; SM= starting material loaded into gel filtration column). Please note that the upper parts of membranes developed with anti-β2-m in B and C were covered during the chemiluminescence signal capture to enhance the specific β2-m signal versus the nonspecific bands due to the secondary antibody as previously shown (1).

REFERENCES

1. Faravelli, G., Raimondi, S., Marchese, L., Partridge, F. A., Soria, C., Mangione, P. P., Canetti, D., Perni, M., Aprile, F. A., Zorzoli, I., Di Schiavi, E., Lomas, D. A., Bellotti,

V., Sattelle, D. B., and Giorgetti, S. (2019) C. elegans expressing D76N β_2-microglobulin: a model for in vivo screening of drug candidates targeting amyloidosis. *Sci Rep* **9**, 19960–19960
